# HMGA1 Activates FGFBP1 Transcription to Enhance Angiogenesis Induction and Tumor Progression via FGF2/FGFR1 Pathway

**DOI:** 10.7150/ijbs.109079

**Published:** 2026-02-26

**Authors:** Zhe Zhang, Yili Xiong, Mingyang Li, Shengyao Wang, Zhenxing Liu, Guanduo Wang, Huanqing Zhang, Xiaojuan Yang, Gangli Liu, Dongsheng Zhang, Haiwei Wu, Shengyun Huang

**Affiliations:** 1 Department of Oral and Maxillofacial Surgery, School and Hospital of Stomatology, Cheeloo College of Medicine, Shandong University & Shandong Key Laboratory of Oral Tissue Regeneration & Shandong Engineering Laboratory for Dental Materials and Oral Tissue Regeneration & Shandong Provincial Clinical Research Center for Oral Diseases, Jinan, Shandong, 250012, China.; 2 Department of Oral and Maxillofacial Surgery, Shandong Provincial Hospital Affiliated to Shandong First Medical University, Jinan, Shandong, 250021, China.; 3 School of Stomatology, Shandong First Medical University, Jinan, Shandong, 250117, China.; 4 Department of Oral and Maxillofacial Surgery, Shandong Provincial Hospital, Cheeloo College of Medicine, Shandong University, Jinan, Shandong, 250021, China.

**Keywords:** HMGA1, FGFBP1, FGF2, FGFR1, Transcription, Angiogenesis

## Abstract

High mobility group AT-hook 1 (HMGA1) is a chromatin regulator overexpressed in various cancers, often predicting poor outcomes. However, its role in head and neck squamous cell carcinoma (HNSCC) remains unclear. A hallmark of HNSCC is the rapid growth of its vasculature. Here, we identify an epigenetic mechanism whereby HMGA1 promotes tumor progression and angiogenesis via upregulation of fibroblast growth factor-binding protein 1 (FGFBP1). *HMGA1* silencing suppressed oncogenic properties *in vitro* and reduced tumor initiating cells in HNSCC xenograft mice. RNA sequencing revealed that HMGA1 regulated transcriptional networks involved in tumor progression and angiogenesis, including the *FGFBP1* gene. HMGA1 directly binds to the *FGFBP1* promoter to induce its expression. This upregulation increased secretion of FGFBP1's target, FGF2. Interestingly, disrupting FGFBP1 via gene silencing or the FGFR1 inhibitor PD166866 recapitulated phenotypes observed with *HMGA1* silencing. Blocking *HMGA1*, *FGFBP1*, or FGFR1 also reduced stromal formation and increased tumor necrosis. In human HNSCC, the combined analysis of HMGA1 and FGFBP1 provides a more detailed evaluation of patient prognosis. Our findings highlight a novel paradigm where HMGA1 and FGFBP1 drive tumor progression and angiogenesis, presenting them as potential therapeutic targets for HNSCC.

## Background

Head and neck squamous cell carcinoma (HNSCC) accounts for about 90% of head and neck malignancies, with poor prognosis and low five-year survival rate, making it a significant public health issue 1. Standard treatments, such as surgery, radiotherapy, chemotherapy, and immunotherapy, offer limited survival improvements, necessitating novel therapeutic approaches 2.

Angiogenesis is one of the hallmarks of HNSCC. Both HNSCC cells and stromal cells produce a variety of pro-angiogenic factors, which activate endothelial cells and induce angiogenesis, serving as a key driver of HNSCC progression 3.

Vascular endothelial growth factor (VEGF) is highly expressed in HNSCC and acts as a pro-angiogenic factor. VEGF and its receptor VEGFR play a dominant role in the angiogenesis system. Multiple inhibitors and other types of drugs targeting VEGF/VEGFR have been approved by the FDA for HNSCC treatment, and numerous drugs are undergoing clinical trials 4. Results from clinical studies over the past 5 years indicate that their combination with chemotherapy, targeted therapy, and immunotherapy enhances the efficacy of these treatments in HNSCC 5-8. This demonstrates the therapeutic potential of anti-angiogenic therapy in HNSCC.

However, due to the complex mechanisms of tumor angiogenesis, anti-angiogenic therapy still faces several shortcomings, including tumor recurrence, drug resistance, lack of biomarkers, short-term efficacy, and severe adverse events 4. Therefore, further investigation into the mechanisms of tumor angiogenesis is necessary for the improvement of anti-angiogenic therapies.

Both HNSCC cells and stromal cells, such as cancer-associated fibroblasts and tumor-associated tissue eosinophils, can induce angiogenesis 9-14. It has been found that HNSCC cells promote angiogenesis through highly expressed phosphofructokinase-platelet (PFKP) or circular RNA circFNDC3B 9,10. Additionally, HNSCC cells can secrete TGFβ^+^ small extracellular vesicles to reprogram primary human macrophages into a pro-angiogenic phenotype 11. This suggests that HNSCC cells can induce angiogenesis through various mechanisms to promote tumor progression. Therefore, studying the mechanisms by which HNSCC cells induce endothelial cell-mediated blood vessel formation is meaningful for a deeper understanding of angiogenesis in HNSCC and may provide new strategies for anti-angiogenic therapy.

Epigenetic regulation, including DNA methylation, histone modifications, chromatin remodeling, and non-coding RNAs, is involved in HNSCC. This can promote the occurrence and development of tumors 15. Therefore, exploring epigenetic phenomena may help to identify novel therapeutic targets. Chromatin modifiers regulate gene expression by altering chromatin structure 16. High mobility group AT-hook 1 (HMGA1) is a chromatin modifier highly expressed in stem cells but low in well-differentiated cells 17. Alternative splicing produces HMGA1a and HMGA1b, which bind to AT-rich DNA regions, open chromatin, and recruit transcriptional regulators and some activated histone modifications, such as acetylated histones 18. HMGA1 is highly expressed in many cancers, often predicting poor prognosis and differentiation 19-25. In pancreatic ductal adenocarcinoma and myeloproliferative neoplasms, HMGA1 silencing reduces tumorigenic properties and prolongs survival in mice models 19,20.

In HNSCC, HMGA1 expression correlates with clinical features like grade and lymph metastasis, though findings vary across studies due to sample and method differences 26-31. In oral potentially malignant diseases, HMGA1 is elevated and activates myofibroblasts 32,33. HMGA1 silencing in breast cancer cells reduces the tube formation of human umbilical vein endothelial cells (HUVECs) by downregulating VEGFA 34. However, its role in HNSCC transcriptional networks and angiogenesis remains unclear.

Our study uncovers a novel role for HMGA1 in HNSCC, showing that it upregulates FGFBP1 to drive angiogenesis and tumor growth. HMGA1 binds to the promoter of fibroblast growth factor-binding protein 1 (*FGFBP1*), enhancing its expression and thereby modulating the fibroblast growth factor 2/ fibroblast growth factor receptor 1 (FGF2/FGFR1) signaling pathway. Silencing *HMGA1* or *FGFBP1* inhibited the oncogenic phenotypes and angiogenesis-inducing ability of HNSCC cells both *in vitro* and *in vivo*. Overexpression of *FGFBP1* or exogenous human FGF2 reversed this effect of *HMGA1* silencing. PD166866, an FGFR1 inhibitor, similarly blocked FGF2 effects, recapitulated the effects of *HMGA1* or *FGFBP1* silencing. High HMGA1 expression correlates with poor prognosis in HNSCC, with subgroups based on HMGA1 and FGFBP1 expression showing differential outcomes. Together, these findings suggest HMGA1/FGFBP1/FGF2/FGFR1 as a promising therapeutic target for HNSCC treatment.

## Methods

### Bioinformatics analysis

scRNA-seq data were processed using “Seurat 5.0,” filtering cells with 500-6000 UMI counts and excluding those with >10% mitochondrial UMIs, resulting in 53,232 cells. PCA and clustering were performed with the top 50 principal components, and nine major cell types were identified. CNVs in epithelial cells were estimated with InferCNV. DEG analysis was done using the "Limma" package (Adjusted *P* < 0.05, |log_2_(si*HMGA1*/control)| ≥ 0.5), and GO enrichment was conducted using “clusterProfiler.” Hallmark gene sets were analyzed via ssGSEA, and Kaplan-Meier survival analyses were performed with “survminer” and “survival.”

### Patient tissue samples

HNSCC tissue samples and matched adjacent non-tumorous tissues were obtained from the Department of Pathology, Shandong Provincial Hospital, collected between May 2023 and March 2024.

### Cell lines and culture conditions

HNSCC cell lines (Cal27, HSC2, SCC15, SCC25), normal human oral epithelial cells (HOEC), and human umbilical vein endothelial cells (HUVECs) were used. HSC2 and HOEC were purchased from Otwo Biotech, Cal27 from Procell, SCC15 and SCC25 from ATCC, and HUVECs from Immocell. All cell lines were authenticated and mycoplasma-negative. Cells were cultured in their respective media with 10% FBS and 1% penicillin-streptomycin, under standard conditions at 37°C with 5% CO₂.

### Cell transfection with siRNA, shRNA and plasmids

siRNAs and plasmids were synthesized by Genomeditech, and lentiviruses by Genechem. Transfection was performed using GMTrans Liposomal Reagent, followed by RT-qPCR and Western blotting 48 hours post-transfection. Cal27 cells were infected with lentivirus for 16 hours, then selected with puromycin (10 μg/mL) for 72 hours to generate stable clones. Silencing or overexpression efficiency was evaluated by RT-qPCR and Western blotting.

### RNA isolation and reverse transcriptase quantitative PCR (RT-qPCR) assay

Total RNA was extracted using the SteadyPure Universal RNA Extraction Kit (Accurate Biology) and cDNA synthesized from 1 µg RNA using the Evo M-MLV RT Mix Kit. RT-qPCR was performed with the 2X SYBR Green Pro Taq HS Premix on the LightCycler 480 system. Gene expression was normalized to β-actin and calculated using the 2^-ΔΔCt method.

### Western blot

Cells were lysed in RIPA buffer, and protein concentration was measured using the BCA assay. Equal protein amounts were separated by SDS-PAGE, transferred to PVDF membranes, and incubated with primary and secondary antibodies. Protein bands were visualized using ECL and imaged with an Amersham Imager 600. Secreted proteins were concentrated with methanol and chloroform and analyzed by Western blotting to assess FGF2 expression.

### Cell proliferation assay

Cell proliferation was assessed using the CCK-8 assay over 4 or 7 days. Cells were seeded in 96-well plates at 3,000 cells per well and incubated at 37°C with 5% CO₂. After treatments, 10 µL of CCK-8 solution was added daily, and absorbance at 450 nm was measured using a ThermoMultiskan GO. Proliferation data were normalized to the control group absorbance on day 0.

### Colony formation assay

Cells were trypsinized, seeded in 6-well plates at 800-1000 cells per well, and cultured for 10-14 days at 37°C with 5% CO₂ until colonies formed. The medium was changed every 3 days. Colonies were fixed with 4% paraformaldehyde, stained with 1% crystal violet, and counted if consisting of 50 or more cells using ImageJ software.

### Scratch assay

HNSCC cells were treated with 10 µM Cytosine β-D-arabinofuranoside (Ara-C) for 1 h to inhibit proliferation, and linear scratches were created in 6-well plates with 1% FBS for 20 hours. Migration was assessed by measuring scratch gaps under a microscope.

### Transwell migration and Matrigel invasion assays

For migration and invasion assays, HNSCC cells were cultured in serum-free medium for 24 hours and then treated with 10 µM Ara-C for 1 hour. Cells (5×10⁴) were seeded in the upper chambers of Transwell inserts, with Matrigel coating for invasion. After 20 hours of incubation, non-migrating/invading cells were removed, and those on the lower surface were fixed, stained with crystal violet, washed, and photographed.

### *In vivo* limiting dilution assay

To evaluate tumor-initiating potential, varying cell numbers (1 × 10^4^, 5 × 10⁴, and 1 × 10^5^) were injected into BALB/c nude mice and monitored for 5, 6, and 9 weeks, respectively. Tumor volume was calculated using the formula: volume = 0.5 × length × width² 35. Tumor-initiating cell frequency was assessed by Extreme Limiting Dilution Analysis (ELDA), with statistical significance determined using the chi-square test 36. Mice were kept in SPF conditions.

### RNA sequencing (RNAseq)

RNA was extracted using RNAex Pro Reagent, and library construction and sequencing were performed by the Beijing Genomics Institute. Data analysis was done using an internal assembler and variant caller. Clean reads were aligned to the reference genome with STAR, and gene read counts were obtained with HTSeq. Differential expression was analyzed using the edgeR package, with genes showing a log_2_ fold change ≥ 0.5 and adjusted P < 0.05 considered significantly different (Supplemental Table 1).

### Ethynyl-2-deoxyuridine (EdU) proliferation assay

Cell proliferation in Cal27 cells was assessed using the EdU Apollo DNA Kit. Transfected cells and controls (3×10³ cells/well) were seeded in 96-well plates and treated with human FGF2 protein. After 72 hours, cells were incubated with 20 μM EdU for 2 hours, fixed, and stained with Apollo 488 for EdU detection and Hoechst 33342 for nuclear staining. EdU-positive cells were quantified using ImageJ software.

### Chromatin immunoprecipitation (ChIP)-quantitative PCR (qPCR) assay

ChIP assays were performed using the Pierce™ Agarose ChIP Kit. Chromatin was cross-linked, sheared, and treated with Micrococcal Nuclease. The FGFBP1 promoter region, predicted to contain an HMGA1 binding site, was amplified using primers designed for a 142 bp product (Supplementary Table 1). After immunoprecipitation, DNA was purified and analyzed by qPCR, with specificity confirmed by gel electrophoresis. Enrichment of the FGFBP1 promoter was calculated relative to a nonspecific IgG control and normalized to input DNA.

### Agarose gel electrophoresis

Agarose gel electrophoresis was performed to assess DNA fragmentation and PCR product specificity. A 1.2% agarose gel was prepared with TAE buffer, stained with nucleic acid dye, and loaded with DNA samples mixed with loading buffer. DNA fragments were sized using a DNA ladder and electrophoresed at 120 V. Bands were visualized using a UV transilluminator.

### Dual-luciferase reporter assay

The dual-luciferase reporter assay was used to assess HMGA1's transcriptional activation of the FGFBP1 promoter. Cal27 cells were co-transfected with firefly luciferase plasmids (wild-type and mutated FGFBP1 promoter regions) and a Renilla luciferase control plasmid. After 48 hours, cell lysates were prepared, and luciferase activities were measured using the Dual-Luciferase Reporter Assay Kit. Firefly luciferase activity was normalized to Renilla activity, and relative luciferase activity was calculated as the ratio of firefly to Renilla luminescence.

### Enzyme-linked immunosorbent assay (ELISA) for human FGF2

FGF2 secretion by Cal27 cells was measured using the Human FGF2 ELISA Kit. Conditioned media or standards (100 µL) were added to a pre-coated 96-well plate and incubated for 2 hours at 37°C. After washing, biotinylated detection antibody and HRP-streptavidin were added. Following substrate addition, absorbance at 450 nm was measured, and FGF2 concentrations were determined from a standard curve and expressed in pg/mL.

### Preparation of conditioned medium

Conditioned medium for ELISA, Western blot, CCK-8, tube formation, and Matrigel plug assays was prepared by culturing Cal27 cells in serum-free DMEM for 24 hours after reaching 80% confluence. The medium was collected, centrifuged at 3,000 rpm for 20 minutes, and stored at -80°C for downstream analysis to assess its effects on HUVECs.

### Transwell co-culture migration assays

Transwell co-culture migration assays were performed in 24-well plates with 8 μm pore size. Cal27 cells were plated in the bottom chamber, and HUVECs (3×10⁴ cells/well) were seeded in the upper chamber in serum-free ECM. After 20 hours of incubation at 37°C with 5% CO₂, migrated HUVECs were stained and photographed.

### Tube formation assay

Matrigel was mixed with Cal27 supernatants in a 1:1 ratio and added to 96-well plates. After 30 minutes, HUVECs (2.5 × 10⁴ cells/well) were seeded and incubated for 1-3 hours. For PD166866 treatment, Matrigel was mixed with DMEM, and HUVECs were treated with PD166866 for 24 hours before seeding in ECM. High-resolution images of the capillary-like structures were captured using an inverted light microscope. Quantitative analysis was performed using the Angiogenesis Analyzer plugin for ImageJ software (NIH). To comprehensively assess the angiogenic ability, the following four parameters were quantified: 1) Number of junctions: the number of branching points connecting segments, indicating the complexity of the vascular network; 2) Number of meshes: the number of enclosed polygonal areas, reflecting the maturity of tube formation; 3) Number of segments: the count of individual linear vessel elements delimited by junctions; 4) Total segments length: the sum of the lengths of all segments, representing the overall extension capability of the endothelial cells.

### Matrigel plug assay

A Matrigel plug assay was performed to assess the effects of Cal27 supernatants on HUVECs. A mixture of 100 μL Matrigel, 100 μL Cal27 supernatants, and 1×10⁶ HUVECs was implanted into nude mice. After two weeks, the mice were euthanized, and the Matrigel plugs were excised for analysis.

### Hematoxylin and eosin (H&E), immunohistochemistry (IHC) and immunofluorescence (IF)

Tissue samples were fixed in 4% paraformaldehyde, dehydrated, cleared in xylene, and embedded in paraffin. Sections (5 µm) were deparaffinized, rehydrated, and stained with hematoxylin and eosin (H&E) for light microscopy imaging. For IHC, sections underwent antigen retrieval, blocking, and incubation with primary and secondary antibodies. Immunoreactivity was detected using a DAB Substrate Kit, and nuclei were counterstained with hematoxylin. Staining intensity, area, and number were quantified using ImageJ software. For immunofluorescence (IF), sections followed the same antigen retrieval and blocking steps, incubated with primary antibodies, and then fluorophore-conjugated secondary antibodies. Nuclei were counterstained with DAPI, and images were captured using a digital slice scanner.

### Multiple immunofluorescence (mIF)

For mIF, 5 µm tissue sections were deparaffinized, rehydrated, and subjected to antigen retrieval in sodium citrate buffer. After blocking with 3% BSA, sections were incubated with primary antibodies (CD31, HMGA1, FGFBP1) overnight at 4°C, followed by secondary antibody incubation and fluorescein-conjugated tyramide signal amplification (TSA). Microwave treatment was applied to remove antibodies, and blocking was repeated. The last primary antibody (anti-FGF2) was incubated overnight, followed by Cy3-conjugated secondary antibody. Nuclei were stained with DAPI, and images were scanned using a digital slice scanner.

### Masson's trichrome staining

Tissue sections were deparaffinized by baking at 65°C for 60 minutes, followed by immersion in xylene and rehydration through graded ethanol. Slides were mordanted with Bouin's solution, stained with Weigert's hematoxylin, and differentiated in acidic ethanol. Sections were stained with Biebrich scarlet-acid fuchsin, treated with phosphomolybdic acid, and stained with aniline blue. After dehydration, clearing in xylene, and mounting with neutral balsam, images were scanned using a digital slice scanner.

### Tumor-bearing mouse model and PD166866 treatment

To establish a tumor-bearing mouse model, 8 × 10⁵ Cal27 cells were injected subcutaneously into BALB/c nude mice. When tumors reached 20-30 mm³, mice were randomized into two groups: PD166866 treatment (30 mg/kg every two days for 30 days) or vehicle control (0.5% sodium carboxymethyl cellulose in saline).

### Statistical analysis

Statistical analyses were performed using GraphPad Prism 9.0. Normality was assessed with the Shapiro-Wilk test, and variances were evaluated using the F test or Bartlett test. For two-group comparisons, a 2-tailed Student's unpaired t-test was used for normally distributed data with equal variances, Welch's correction for unequal variances, and the Mann-Whitney test for non-normally distributed data. For multiple groups, one-way ANOVA with Dunnett's multiple comparisons test was applied for normally distributed data, and the Kruskal-Wallis test for non-normally distributed data. Two-way ANOVA with Šídák's test was used for two-factor comparisons. Survival was analyzed by the log-rank test, and correlation was assessed using Pearson's correlation coefficients. A p-value < 0.05 was considered statistically significant. Data are presented as mean ± SD or SEM.

## Results

### HMGA1 is highly expressed and is associated with poor prognosis in HNSCC patients

**Figure 1:**
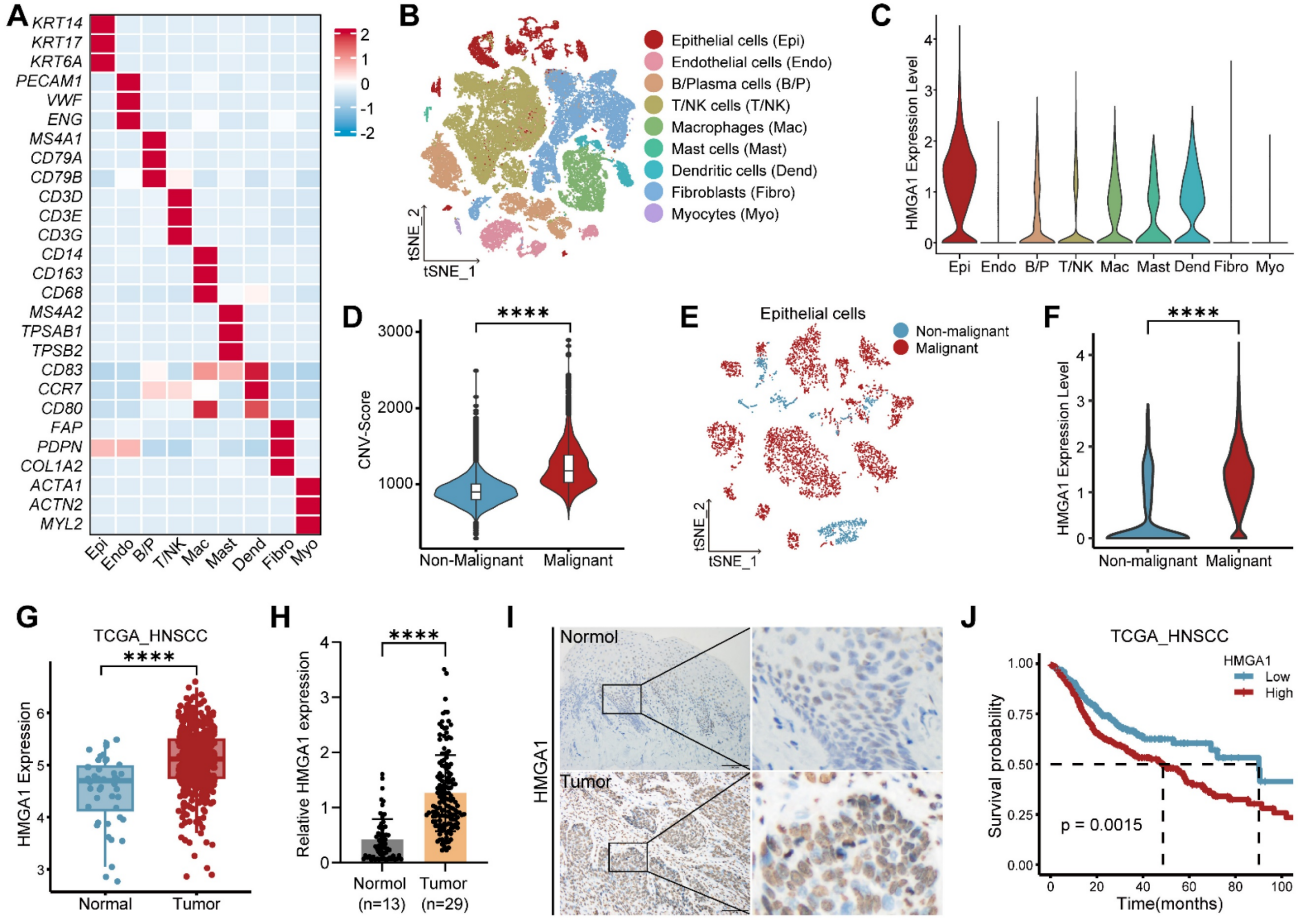
** HMGA1 is highly expressed and is associated with poor prognosis in HNSCC. (A)** Heatmap of signature genes for distinct cell types, which are represented by three specifically expressed genes. **(B)** The tSNE plot of the 53,232 cells delineates their cell types, with distinct colors distinguishing cell types. **(C)** Violin plot showing expression of HMGA1 in distinct cell types. **(D)** Violin plot showing CNV scores between non-malignant cells and malignant cells. **(E)** The tSNE plot showing the distribution of non-malignant epithelial cells (blue) and malignant epithelial cells (red). **(F)** Violin plot showing expression of HMGA1 between non-malignant epithelial cells (blue) and malignant epithelial cells (red). Data **(A-F)** are form scRNA-seq (GSE181919). **(G)** Boxplot showing expression of HMGA1 between normal (n=44 patients) and tumor (n=516 patients) in the TCGA-HNSCC cohort. **(H, I)** IHC showing the expression of HMGA1 in adjacent tissues (n=13 patients) and tumors (n=29 patients) with HNSCC. Scale bars: 100μm. **(J)** The Kaplan-Meier overall survival curves between low-HMGA1 group (n=177 patients) and high-HMGA1 group (n=339 patients) of TCGA-HNSCC cohort. Results are shown as mean ± standard deviation (SD). ****P<0.0001; 2-tailed Student's unpaired *t*-test **(D, F and G)**, Mann-Whitney test **(H)**, log-rank test **(J)**.

To assess HMGA1 expression across multiple cancers, we analyzed data from GEPIA, showing elevated HMGA1 in various cancer types compared to normal tissues** (Fig. S1A)**. In HNSCC, single-cell RNA-seq data (GSE181919) identified nine major cell types, with epithelial cells showing notably higher HMGA1 expression **(Fig. 1A-C)**. Using inferCNV, we distinguished malignant from non-malignant epithelial cells, finding HMGA1 significantly elevated in malignant cells **(Figs. S1B and 1D-F)**. Consistent results from TCGA-HNSCC and GEO cohorts confirmed higher HMGA1 expression in HNSCC than adjacent normal tissues **(Figs. 1G and S1C-E)**. Immunohistochemistry (IHC) of HNSCC tissues (n=29) and adjacent normal tissues (n=13) further validated this finding **(Fig. 1H and I)**. Additionally, higher HMGA1 levels correlated with shorter overall survival (OS) in HNSCC patients **(Figs. 1J and S1F)**, suggesting HMGA1's link to poor prognosis.

### *HMGA1* silencing disrupts oncogenic properties of HNSCC *in vitro* and *in vivo*

**Figure 2:**
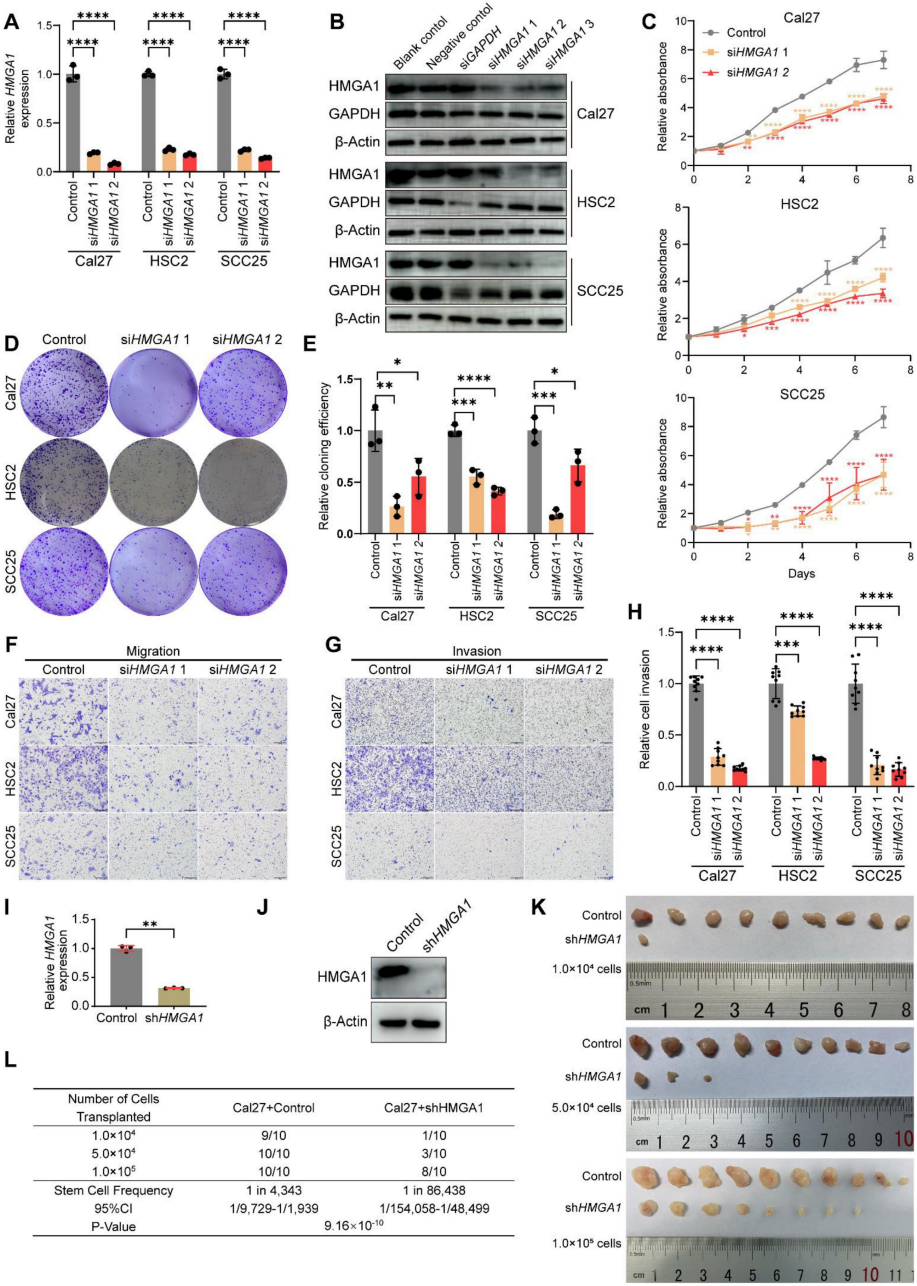
***HMGA1* silencing disrupts oncogenic properties of HNSCC *in vitro* and *in vivo*. (A)** qRT-PCR showing the expression of HMGA1 mRNA in HNSCC cell lines (Cal27, HSC2, SCC25) with and without *HMGA1* silencing via siRNA targeting 2 different sequences (siRNA1, siRNA2) from 3 experiments performed in triplicate. **(B)** Western blot (*n* = 3 independently biological experiments) showing the expression of HMGA1 protein in HNSCC cells with and without *HMGA1* silencing. **(C)** CCK-8 assay showing the proliferation ability of HNSCC cells with and without *HMGA1* silencing from 3 experiments performed in triplicate. **(D, E)** Clonogenic assay showing the clonogenic ability of HNSCC with and without *HMGA1* silencing from 3 experiments performed in triplicate. **(F)** Transwell migration assay showing the migration ability of HNSCC cells with and without *HMGA1* silencing following treatment with 10μM cytosine β-D-arabinoside (Ara-C) for 1 hour to mitigate effects of proliferation from 3 experiments performed in triplicate. Scale bars: 200μm. **(G, H)** Matrigel invasion assay showing the invasive ability of HNSCC cells with and without *HMGA1* silencing following treatment with 10μM Ara-C for 1 hour from 3 experiments performed in triplicate. Scale bars: 200μm.** (I, J)** qRT-PCR and western blot (*n* = 3 independently biological experiments in **I** and **J**) showing the expression of HMGA1 mRNA and protein in Cal27 cells with and without *HMGA1* silencing via lentiviral delivery of shRNA targeting the same sequence as si*HMGA1* 1. **(K)** *In vivo* limiting dilution assay showing xenograft tumorigenicity of Cal27 cells with and without *HMGA1* silencing at different limiting dilutions (n=10/condition). **(L)** Extreme limiting dilution analysis (ELDA) showing the frequency of tumor initiator cells. Data show 95% confidence interval around tumor initiator cell frequency **(L)**. Results are shown as mean ± SD **(A, C, E, H, I)**. *P<0.05, **P<0.01, ***P<0.001, ****P<0.0001; ordinary 1-way ANOVA with Dunnett's multiple comparisons test **(A, C, E)**, Brown-Forsythe and Welch's ANOVA test with Dunnett's T3 multiple-comparisons test **(H)**. 2-tailed Student's unpaired* t*-test with Welch's correction **(I)**, chi-square test **(L)**.

We found that HMGA1 expression is higher in HNSCC cell lines (Cal27, SCC25, HSC2 and SCC15) compared to normal human oral epithelial cells (HOEC) **(Fig. S2A and B)**. To explore HMGA1 function, we selected two si*HMGA1* sequences to reduce HMGA1 mRNA and protein levels **(Fig. 2A and B)**. CCK8, colony formation, scratch, transwell and Matrigel assays revealed that *HMGA1* silencing inhibits proliferation, clonogenicity, migration, and invasion in HNSCC cells, underscoring its role in these oncogenic behaviors **(Figs. 2C-H and S2C-E)**. Next, we investigated the role of HMGA1 in tumorigenesis *in vivo*. We constructed a short hairpin RNA (shRNA) based on the sequence of si*HMGA1* 1 and successfully silenced *HMGA1* in Cal27 by lentiviral delivery **(Fig. 2I, J)**. Using ELDA assay 36, we found that *HMGA1* deficiency reduced the number, volume, weight of tumors as well as tumor-initiating cells, confirming HMGA1's importance in tumorigenesis **(Figs. 2K, L and S2F, G)**.

### HMGA1 regulates carcinogenic transcriptional network and induces FGFBP1 expression

**Figure 3:**
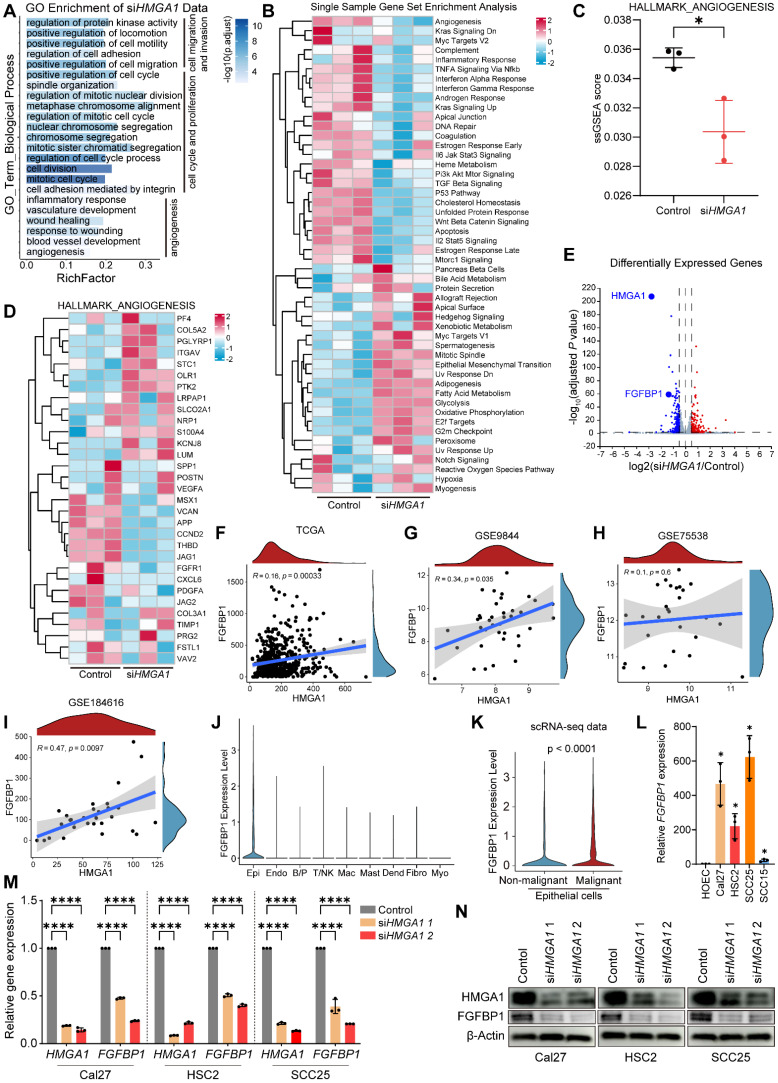
** HMGA1 regulates carcinogenic transcriptional network and induces FGFBP1 expression. (A)** GO analysis of differentially expressed genes (DEGs) in RNA-seq data of Cal27 cells with and without *HMGA1* silencing.** (B)** Single sample gene set enrichment analysis (ssGSEA) showing the different enrichment of DEGs for different gene sets (MSigDb Hallmark) in Cal27 cells with and without *HMGA1* silencing. The colors are encoded by values of the ssGSEA scores in different samples. **(C)** ssGSEA showing the differential enrichment of DEGs for gene sets associated with angiogenesis (MSigDb Hallmark) in Cal27 cells with and without *HMGA1* silencing.** (D)** ssGSEA showing the different enrichment of genes in gene sets associated with angiogenesis (MSigDb Hallmark) in Cal27 cells with and without *HMGA1* silencing. **(E)** Volcano plot showing DEGs (1123 up- and 1375 downregulated) in Cal27 with and without *HMGA1* silencing and *FGFBP1* among the genes most repressed with *HMGA1* silencing. Thresholds are shown as dashed lines. Adjusted *P* < 0.05, |log_2_(si*HMGA1*/control)| ≥ 0.5. **(F)** Correlation analysis of HMGA1 and FGFBP1 expression in TCGA-HNSCC cohort. 95% confidence interval (CI) is indicated with gray color. **(G)** Correlation analysis of HMGA1 and FGFBP1 expression in GSE9844 cohort. 95% confidence interval (CI) is indicated with gray color.** (H)** Correlation analysis of HMGA1 and FGFBP1 expression in GSE75538 cohort. 95% confidence interval (CI) is indicated with gray color.** (I)** Correlation analysis of HMGA1 and FGFBP1 expression in GSE184616 cohort. 95% confidence interval (CI) is indicated with gray color. **(J)** Violin plot showing expression of FGFBP1 in distinct cell types.** (K)** Violin plot showing expression of FGFBP1 between non-malignant epithelial cells (blue) and malignant epithelial cells (red). Data (**J, K**) are form scRNA-seq (GSE181919). **(L)** qRT-PCR showing the expression of FGFBP1 mRNA in HNSCC cell lines (Cal27, HSC2, SCC25, SCC15) from 1 experiment performed in triplicate. **(M)** qRT-PCR showing the expression of HMGA1 and FGFBP1 mRNA in HNSCC cells (Cal27, HSC2, SCC25) with and without *HMGA1* silencing from 3 experiments performed in triplicate. **(N)** Western blot (*n* = 3 independently biological experiments) showing the expression of HMGA1 and FGFBP1 protein in HOEC and HNSCC cells with and without *HMGA1* silencing. Data shown as mean ± SD. *P<0.05, ****P<0.0001; 2-tailed Student's unpaired* t*-test **(C, K)**, 2-tailed Student's unpaired* t*-test with Welch's correction** (L)**, ordinary 1-way ANOVA with Dunnett's multiple comparisons test **(M)**.

To further investigate the mechanism of HMGA1, a regulator at the transcriptional level, we performed RNA sequencing in Cal27 cells with or without *HMGA1* silencing. Gene ontology (GO) enrichment analysis revealed an HMGA1 signature of genes involved in biological processes related to tumor progression, such as cell cycle and proliferation, cell migration and invasion, and especially, angiogenesis **(Fig. 3A)**. Single sample gene set enrichment analysis (ssGSEA, MSigDb Hallmark gene sets) identified great diversity of the sample activity in 50 hallmark gene sets **(Fig. 3B)**. Unexpectedly, angiogenesis pathway is also enriched **(Fig. 3C, D)**. This suggests the potential effect of HMGA1 on angiogenesis in HNSCC. Differentially expressed genes (DEGs) (adjusted *P* < 0.05, |log_2_(si*HMGA1*/control)| ≥ 0.5) included 190 up- and 207 downregulated genes **(Fig. 3E, listed in Supplemental Table 3)**.

Interestingly, our data showed that the expression of fibroblast growth factor-binding protein 1 (*FGFBP1*) was reduced after silencing *HMGA1* **(Fig. 3E)**, and FGFBP1 is also involved in angiogenesis 37. In addition, HMGA1 and FGFBP1 correlated positively **(Fig. 3F-I)**. Single-cell RNA-seq data (GSE181919) showed that FGFBP1 was most expressed in epithelial cells and was higher in malignant than nonmalignant epithelium **(Fig. 3J, K)**. Consistent with this, FGFBP1 expression was also higher in HNSCC cell lines than in HOEC **(Fig. 3L)**. Therefore, we first focused on FGFBP1. We further found that silencing *HMGA1* reduced FGFBP1 expression (mRNA, protein) **(Fig. 3M, N)**. These results provide preliminary evidence for a link between HMGA1 and FGFBP1.

### Silencing *FGFBP1* recapitulates effects of silencing *HMGA1*

**Figure 4:**
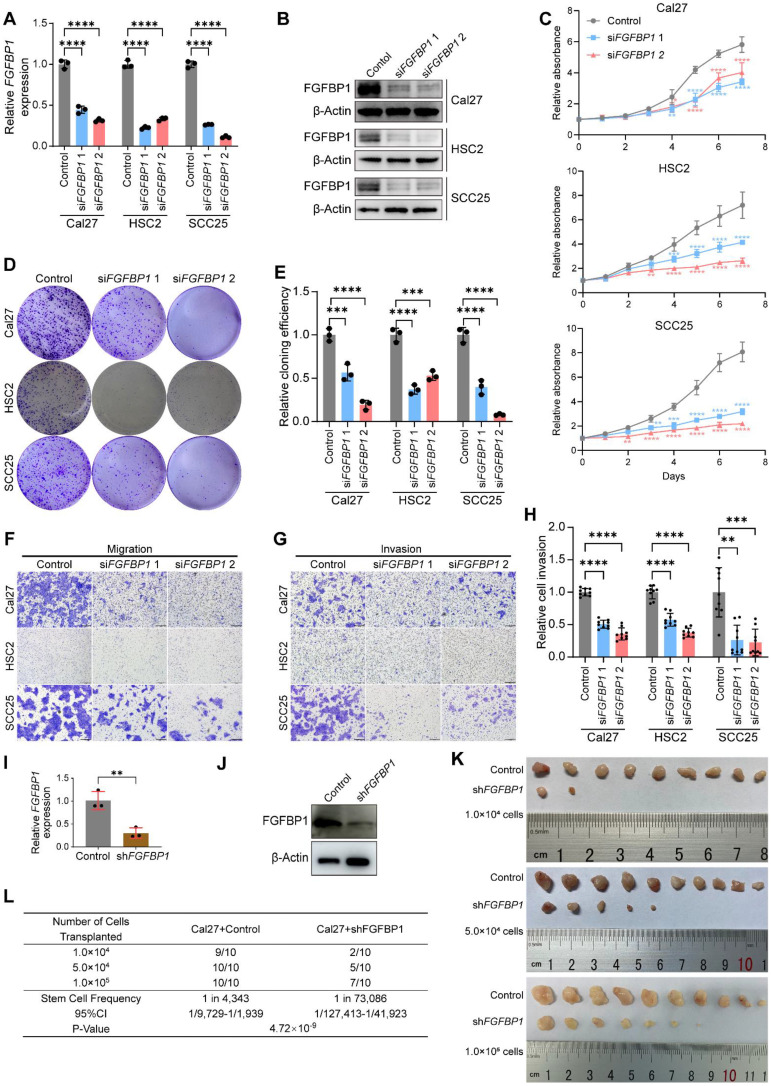
***FGFBP1* silencing recapitulates effects of *HMGA1* silencing in HNSCC *in vitro* and *in vivo*. (A)** qRT-PCR showing the expression of FGFBP1 mRNA in HNSCC cell lines (Cal27, HSC2, SCC25) with and without *FGFBP1* silencing via siRNA targeting 2 different sequences (siRNA1, siRNA2) from 3 experiments performed in triplicate. **(B)** Western blot (*n* = 3 independently biological experiments) showing the expression of FGFBP1 protein in HNSCC cells with and without *FGFBP1* silencing. **(C)** CCK-8 assay showing the proliferation ability of HNSCC cells with and without *FGFBP1* silencing from 3 experiments performed in triplicate. **(D, E)** Clonogenic assay showing the clonogenic ability of HNSCC with and without *FGFBP1* silencing from 3 experiments performed in triplicate. **(F)** Transwell migration assay showing the migration ability of HNSCC cells with and without *FGFBP1* silencing following treatment with 10μM cytosine β-D-arabinoside (Ara-C) for 1 hour to mitigate effects of proliferation from 3 experiments performed in triplicate. Scale bars: 200μm. **(G, H)** Matrigel invasion assay showing the invasive ability of HNSCC cells with and without *FGFBP1* silencing following treatment with 10μM Ara-C for 1 hour from 3 experiments performed in triplicate. Scale bars: 200μm. **(I, J)** qRT-PCR and western blot (*n* = 3 independently biological experiments in **I** and **J**) showing the expression of FGFBP1 mRNA and protein in Cal27 cells with and without *FGFBP1* silencing via lentiviral delivery of shRNA targeting the same sequence as si*FGFBP1* 2. **(K)** *In vivo* limiting dilution assay showing xenograft tumorigenicity of Cal27 cells with and without *FGFBP1* silencing at different limiting dilutions (n=10/condition). **(L)** ELDA showing the frequency of tumor initiator cells. Data show 95% confidence interval around tumor initiator cell frequency **(L)**. Data shown as mean ± SD **(A, C, E, H, I)**. *P<0.05, **P<0.01, ***P<0.001, ****P<0.0001; ordinary 1-way ANOVA with Dunnett's multiple comparisons test **(A, C, E, Cal27 and HSC2 of H)**, Kruskal-Wallis test with Dunn's multiple-comparison test **(SCC25 of H)**. 2-tailed Student's unpaired* t*-test **(I)**, chi-square test **(L)**.

We further investigated the role of FGFBP1 in HNSCC. First, we successfully silenced *FGFBP1* (mRNA and protein) in HNSCC cell lines **(Fig. 4A, B)**. We found that FGFBP1 deficiency recapitulated the inhibitory effect of *HMGA1* silencing on HNSCC. At the cellular level *in vitro*, FGFBP1 deficiency inhibited proliferation, clonogenicity, migration, and invasion, consistent with the findings of Shintani et al 38 **(Figs. 4C-H and S3A-C)**.

Using 5-ethynyl-2′-deoxyuridine (EdU) experiments, we demonstrated that silencing *HMGA1* or *FGFBP1* inhibited DNA synthesis in HNSCC cells, and overexpression of *FGFBP1* or addition of human FGF2 protein reversed this inhibition, suggesting that FGFBP1 and FGF2 mediates the proliferative effect of HMGA1 **(Fig. S3D-G)**.

Next, we successfully constructed Cal27 cells with stable *FGFBP1* silencing by lentiviral delivery using shRNA **(Fig. 4I, J)**. We used xenograft mice to investigate the effect of FGFBP1 on tumorigenesis ability, and simultaneously performed ELDA analysis 36. Like *HMGA1* silencing, FGFBP1 deficiency inhibited tumorigenesis and reduced the proportion of tumor-initiating cells **(Figs. 4K, L and S3H, I)**.

### HMGA1 activates *FGFBP1* transcription by binding to its promoter

**Figure 5:**
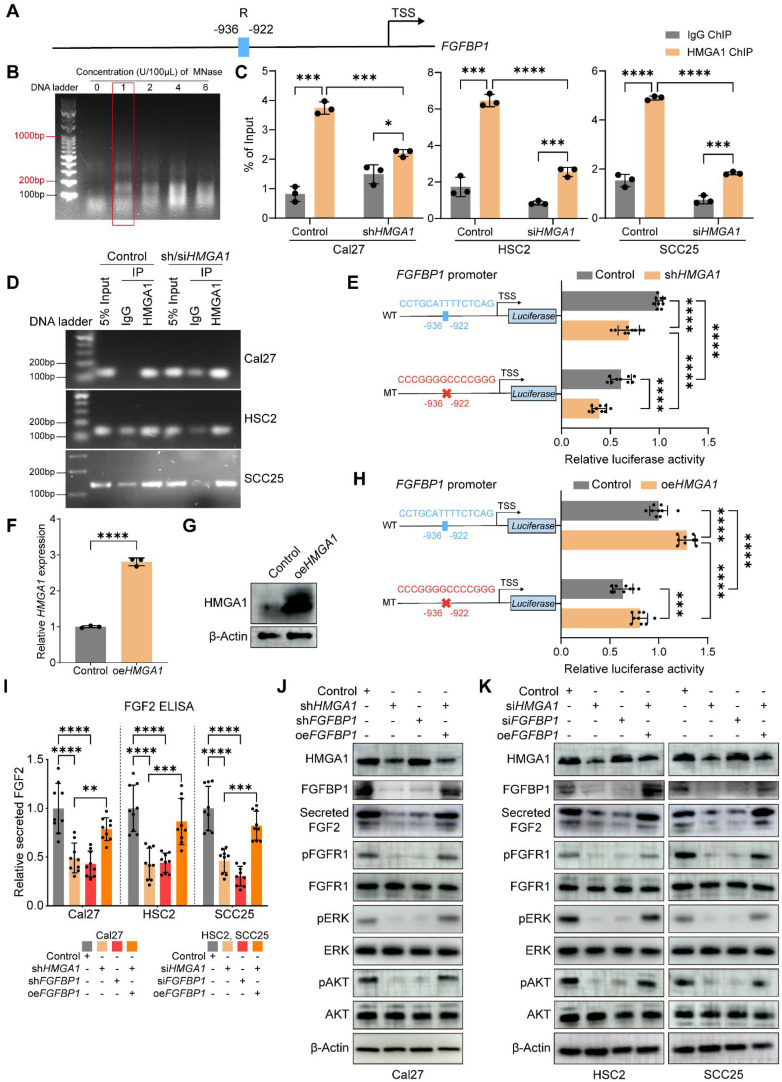
** HMGA1 activates *FGFBP1* transcription by binding to its promoter and alters FGFBP1/FGF2/FGFR1 pathways. (A)** Schematic representation of the *FGFBP1* promoter; R is the predicted HMGA1 binding site. **(B)** The extracted genomic DNA is sheared into smaller, workable pieces enzymatically by digestion with different concentrations of micrococcal nuclease (MNase). 1U/100μL is the optimal concentration which yields more fragments from 200 to 1000 base pairs. **(C, D)** ChIP-qPCR and agarose gel electrophoresis showing HMGA1 occupancy on the *FGFBP1* promoter in HNSCC cells (Cal27, HSC2, SCC25) with and without *HMGA1* silencing. **(E)** Dual-luciferase reporter gene assay showing the transcriptional activation ability of HMGA1 on *FGFBP1* promoter in Cal27 cells transfected with *HMGA1* silencing or control vector and *FGFBP1* promoter constructs including wild type and mutant type. The potential HMGA1 binding site (-936/-922) is mutated from CCTGCATTTTCTCAG to CCCGGGGCCCCGGG. **(F, G)** qRT-PCR and western blot (*n* = 3 independently biological experiments in **F** and **G**) showing the expression of HMGA1 mRNA and protein in Cal27 cells with and without HMGA1 overexpression via plasmid delivery.** (H)** Dual-luciferase reporter gene assay showing the transcriptional activation ability of HMGA1 on *FGFBP1* promoter in Cal27 cells transfected with *HMGA1* overexpression or control vector and *FGFBP1* promoter constructs. **(I)** ELISA showing the expression of secreted FGF2 in HNSCC cells under different conditions from 3 experiments performed in triplicate. **(J, K)** Western blot (*n* = 3 independently biological experiments) showing the expression of secreted FGF2, FGFR1 and its downstream signaling molecules (ERK, AKT), including total and phosphorylated proteins in HNSCC cells under different conditions (control, *HMGA1* silencing, *FGFBP1* silencing, *HMGA1* silencing + *FGFBP1* overexpressing). shRNA is used to silence *HMGA1* and *FGFBP1* in Cal27, while siRNA is used in HSC2 and SCC25. Data shown as mean ± SD. ***P<0.001, ****P<0.0001; 2-tailed Student's unpaired* t*-test **(C, MT (sh*HMGA1* control), sh*HMGA1* (WT MT), F, H)**, 2-tailed Student's unpaired* t*-test with Welch's correction **(WT(sh*HMGA1* control) and Control(WT MT))**, ordinary 1-way ANOVA with Dunnett's multiple comparisons test **(I).**

HMGA1 is a transcriptional regulator by binding to noncoding regions on DNA. To further explore the link between HMGA1 and FGFBP1, first, we predict that HMGA1 has a binding site on the promoter of *FGFBP1* by using the online website hTFtarget. This site is located from -936 to -922 base pairs upstream of the transcription start site (TSS) of *FGFBP1*, and we designated it R **(Fig. 5A)**. First, we determined the optimal concentration (1U/100μL) of Micrococcal Nuclease (MNase) in HNSCC cells **(Fig. 5B)**. A pair of primers were designed for this site, and the length of the product was 142 bp. Next, we performed ChIP-qPCR and agarose gel electrophoresis and found that the proportion of DNA fragments containing this site in the silencing group was less than that in the control group, which was verified in all three HNSCC cells **(Fig. 5C and D)**. Thus, we demonstrated that HMGA1 can bind directly to the promoter of *FGFBP1*.

To investigate whether HMGA1 transcriptionally activates *FGFBP1*, we further performed a dual luciferase reporter gene assay. The wild-type promoter of *FGFBP1* was constructed upstream of the luciferase gene. Compared with the control group, the luciferase expression in the silencing HMGA1 group was lower, indicating that HMGA1 transcriptionally activates *FGFBP1*. To investigate whether HMGA1 binding site R is a functional site, we next mutated this site (5'-CCTGCATTTTCTCAG-3' to 5'-CCCGGGGCCCCGGGG-3'). The mutant promoter was constructed upstream of the luciferase gene. Compared with the wild type, the expression of luciferase in the mutant group was lower, indicating that this site is also a functional site. In addition, after silencing *HMGA1*, the luciferase expression was still decreased in the mutant group, indicating that there are other functional binding sites besides this site **(Fig. 5E)**. These results were also demonstrated by overexpression of *HMGA1* **(Fig. 5F-H)**.

In summary, we have identified a deep molecular biological connection between HMGA1 and FGFBP1. HMGA1 directly binds to the promoter of *FGFBP1* and transcriptionally activates it.

### HMGA1 alters FGFBP1/FGF2/FGFR1 and downstream signals

FGFBP1, a molecular chaperone of the fibroblast growth factor 2 (FGF2), increases the extracellular secretion of FGF2 37,39. FGF2 binds the fibroblast growth factor receptor 1 (FGFR1) to activate downstream signaling pathways, such as ERK/AKT 40. Therefore, we hypothesized that HMGA1 could increase the expression of FGFBP1 to promote the secretion of FGF2 and further activate the downstream signaling pathway of FGFR1. To test this hypothesis, we found that silencing *HMGA1* or *FGFBP1* reduced the secretion of FGF2 in the supernatant (WB and ELISA), and overexpression of *FGFBP1* reversed this decrease **(Fig. 5I-K)**. In addition, various downstream phosphorylated effector proteins were reduced after silencing *HMGA1* or *FGFBP1*, including p-FGFR1, p-ERK1/2, and p-AKT **(Fig. 5J, K)**. Therefore, we can reasonably assume that HMGA1 exerts its biological functions at least in part through FGFBP1/FGF2/FGFR1 pathway.

### HMGA1 enables HNSCC cells to induce HUVECs to form vessels via FGFBP1

Studies suggest HMGA1 and FGFBP1 promote angiogenesis, but their role in HNSCC remains unclear. We investigated this via several experiments. Our RNA-seq data indicated HMGA1-regulated genes are enriched in angiogenic pathways **(Fig. 3A-D)**. FGFBP1 correlates with microvessel density in HNSCC tumors 41,42. These suggest that HMGA1 and FGFBP1 may promote angiogenesis in HNSCC.

**Figure 6:**
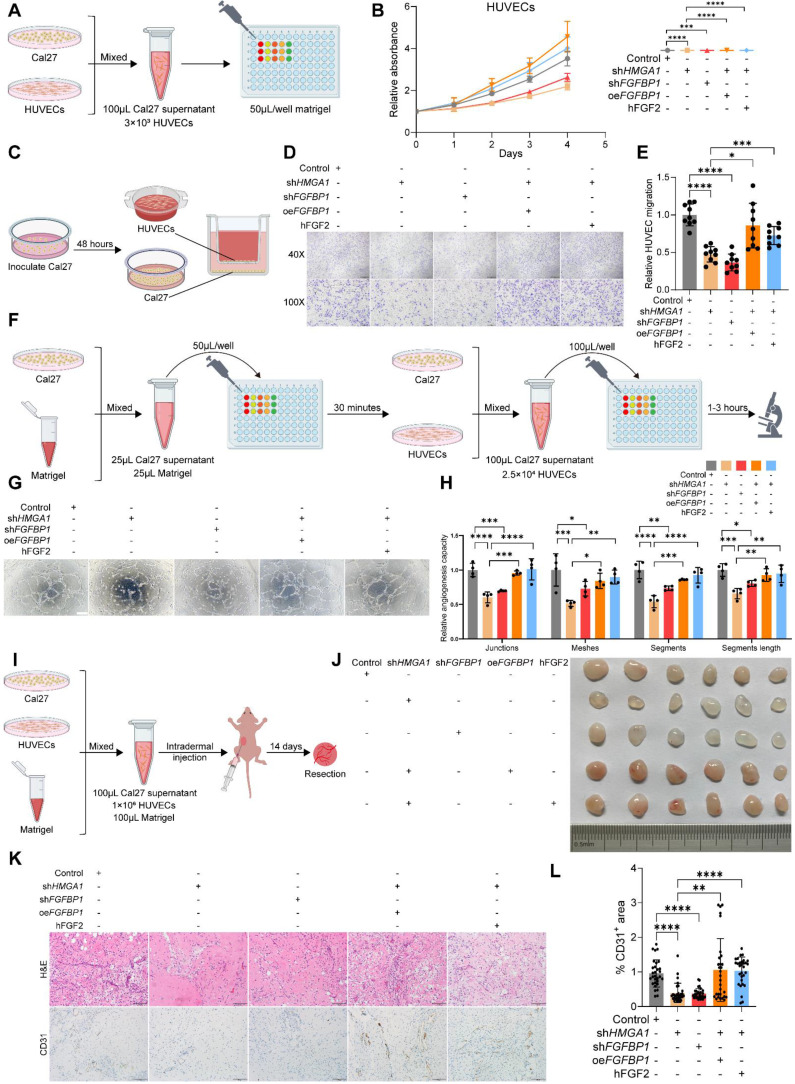
** HMGA1 enables HNSCC cells to induce HUVECs to form vessels via FGFBP1. (A, B)** CCK-8 showing the proliferation ability of HUVECs treated with different conditioned supernatants of Cal27 cells (control, *HMGA1* silencing, *FGFBP1* silencing, *HMGA1* silencing + *FGFBP1* overexpressing, *HMGA1* silencing + 100ng/mL hFGF2) from 3 experiments performed in triplicate. **(C-E)** Transwell co-culture assay showing the migration ability of HUVECs co-cultured with Cal27 cells under different conditions from 3 experiments performed in triplicate. Scale bar, ×40 magnification, 500 μm; scale bar, ×100 magnification, 200 μm. **(F-H)** Tube formation assay (n=4 per condition) showing the *in vitro* angiogenic ability of HUVECs treated with different conditioned supernatants of Cal27 cells. Scale bar, 200 μm. **(I, J)** Matrigel plug assay (n=6 per condition) showing the *in vivo* angiogenic ability of HUVECs treated with different conditioned supernatants of Cal27 cells (control, *HMGA1* silencing, *FGFBP1* silencing, *HMGA1* silencing + *FGFBP1* overexpressing, *HMGA1* silencing + 100ng/mL hFGF2).** (K, L)** % CD31^+^ area of the IHC for CD31and cell nucleus of the Matrigel plug showing the *in vivo* angiogenic ability of HUVECs treated with different conditioned supernatants of Cal27 cells (control, *HMGA1* silencing, *FGFBP1* silencing, *HMGA1* silencing + *FGFBP1* overexpressing, *HMGA1* silencing + 100ng/mL hFGF2). Scale bar, 100 μm. Data from 5 fields at ×20 magnification of tumors from 6 different mice per condition. Data shown as mean ± SD. *P<0.05, **P<0.01, ***P<0.001, ****P<0.0001; 2-way ANOVA with Šídák's multiple comparisons test **(B)**, Brown-Forsythe and Welch's ANOVA test with Dunnett's T3 multiple-comparisons test **(E)**, ordinary 1-way ANOVA with Dunnett's multiple comparisons test **(H)**. Kruskal-Wallis test with Dunn's multiple-comparison test **(L)**.

First, CCK8 assays revealed that supernatant from Cal27 cells with *HMGA1* or *FGFBP1* silencing reduced HUVECs' proliferation, while *FGFBP1* overexpression and added FGF2 (a pro-angiogenic factor) reversed this inhibition **(Fig. 6A, B)**. Transwell co-cultures confirmed silencing *HMGA1* or *FGFBP1* inhibited Cal27-induced HUVECs' migration, which was reversed by *FGFBP1* overexpression or FGF2 **(Fig. 6C-E)**. *In vitro* tube formation assays with Cal27-conditioned medium showed similar trends **(Fig. 6F-H)**, which were quantitatively confirmed by analyzing four key parameters: the number of junctions, the number of meshes, the number of segments, and the total segments length (see the “Tube formation assay” section in Methods for details). We can reasonably speculate that silencing *HMGA1* or silencing *FGFBP1* reduces secretion of components that promote vascular endothelial proliferation and angiogenesis, while overexpression of *FGFBP1* released more FGF2 and reversed this reduction.

Next, a Matrigel plug assay in nude mice indicated that *HMGA1* or *FGFBP1* silencing produced lighter plugs, while *FGFBP1* overexpression or added FGF2 turned them redder **(Fig. 6I, J)**. CD31 IHC showed lower CD31 in *HMGA1* or *FGFBP1* silencing groups, but higher levels with *FGFBP1* overexpression or added FGF2 **(Fig. 6K-M)**. The above results were again confirmed by IF staining of typical Matrigel plugs with DAPI and CD31 **(Fig. S4A)**. These results demonstrate that HMGA1 and FGFBP1 enable HNSCC cells to induce endothelial angiogenesis.

### *HMGA1* or *FGFBP1* silencing reduces stromal formation, inhibits angiogenesis and causes tumor necrosis in HNSCC xenografts

**Figure 7:**
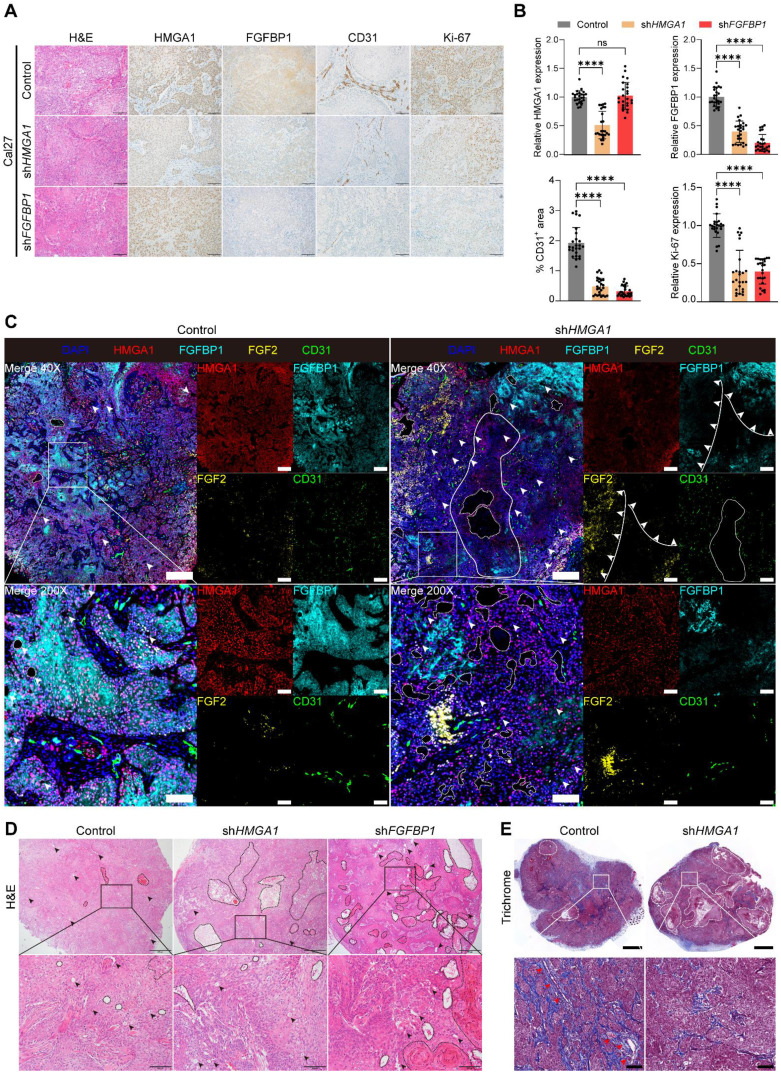
***HMGA1* or *FGFBP1* silencing reduces stromal formation, inhibits angiogenesis and causes tumor necrosis in HNSCC xenografts. (A, B)** H&E staining and IHC for HMGA1, FGFBP1, CD31, Ki67 in Cal27 xenografts with and without *HMGA1* or *FGFBP1* silencing. Data from 5 fields at ×200 magnification of tumors from 5 different mice per condition. Scale bar, 100 μm.** (C)** Representative images of mIF for HMGA1, FGFBP1, FGF2, CD31 in Cal27 xenografts with and without *HMGA1* or *FGFBP1* silencing. Dashed circles and arrows: cell-free regions; solid circle: avascular region; solid lines plus arrows: regions with low or high expression of FGFBP1 and FGF2. Scale bar, 500 μm; scale bar, local magnification, 100μm. **(D)** Representative images of H&E staining in Cal27 xenografts with and without *HMGA1* or *FGFBP1* silencing. Dashed circles and arrows: cell-free and necrotic regions. Scale bar, 500 μm; scale bar, local magnification, 100μm. **(E)** Representative images of Masson's trichrome staining in Cal27 xenografts with and without *HMGA1* silencing. Dashed circles: stroma-free and cell-free regions; red arrows: vessels. Scale bar, 1 mm; scale bar, local magnification, 100μm. Control group and sh*HMGA1* group in (**C**, **D** and **E**) were from the same tumor, respectively. Data shown as mean ± SD. ns, not significant; ****P<0.0001; Kruskal-Wallis test with Dunn's multiple-comparison test **(B)**.

IHC on xenograft tumors showed reduced CD31 levels in *HMGA1* or *FGFBP1* silencing groups, indicating decreased angiogenesis **(Fig. 7A, B)**. IF results supported these findings** (Fig. S4B)**. Additionally, Ki67-positive cells decreased, reflecting reduced proliferation, while *HMGA1* silencing lowered FGFBP1 expression **(Fig. 7A, B)**. Some regions in the sh*HMGA1* group still showed high HMGA1 levels, suggesting silencing escape may be necessary for tumor formation, aligning with previous studies 19.

Multiple immunofluorescence (mIF) staining** (Fig. 7C)** revealed decreased HMGA1 and FGFBP1 fluorescence in the sh*HMGA1* group. Nuclear FGF2 was higher in the sh*HMGA1* group, with a negative correlation between FGFBP1 and FGF2 fluorescence (**Fig. 7C**, solid lines and arrows), indicating reduced FGFBP1 leads to nuclear retention and decreased secretion of FGF2, consistent with Frank et al. 37. Persistent high FGFBP1 expression in some regions suggests escape from *HMGA1* silencing. CD31 levels were lower, reflecting reduced angiogenesis.

More cell-free areas were observed in the sh*HMGA1* group than in the control (**Fig. 7C**, dashed circles and arrows), suggesting increased tumor necrosis due to *HMGA1* silencing. H&E staining confirmed these necrotic areas, which lacked normal cellular structure and nearby blood vessel formation (**Fig. 7D**, dashed circles and arrows). No blood vessels were observed near two large cell-free regions in the sh*HMGA1* group (**Fig. 7C**, the solid circle). This indicates that *HMGA1* silencing inhibits angiogenesis, leading to tumor necrosis. Similarly, sh*FGFBP1* tumors showed more necrotic areas compared to controls (**Fig. 7D**, dashed circles and arrows), supporting that *FGFBP1* silencing also reduces angiogenesis and causes necrosis.

mIF results indicate reduced tumor stroma in the sh*HMGA1* group compared to the control** (Fig. 7C)**, confirmed by Masson trichrome staining (**Fig. 7E**, dashed circles), consistent with Lionel et al. 19. Stroma was absent near necrotic areas in the sh*HMGA1* group (**Fig. 7E**, dashed circles), while blood vessels formed in the control stroma (**Fig. 7E**, red arrows). As tumor stroma supports angiogenesis, *HMGA1* silencing likely reduces stroma and blood vessel formation, leading to necrosis and inhibited tumor growth.

### HMGA1 and FGFBP1 together predict the prognosis of HNSCC patients

Pan-cancer analysis (GEPIA) showed FGFBP1 expression varied across tumors** (Fig. S4A)**. RNA-seq data (TCGA-HNSCC, GEO) revealed no significant differences between HNSCC and controls **(Fig. S5B-E)**. Begum et al. and Li et al. reported high FGFBP1 expression in HNSCC 41,42, while Bem et al. found the opposite 43. IHC showed lower FGFBP1 expression in tumors compared to adjacent margins **(Fig. S5F, G)**. FGFBP1 expression varied within tumors, suggesting its potential prognostic role. Then, we found higher FGFBP1 levels correlated with shorter overall survival (OS) in HNSCC patients **(Fig. S5H)**. We divided patients into four subgroups based on HMGA1 and FGFBP1 expression. Notably, high FGFBP1 expression predicted worse prognosis in the HMGA1 low group, while low FGFBP1 expression predicted worse outcomes in the HMGA1 high group, aiding HNSCC prognosis assessment **(Fig. S5J)**.

### FGFR1 inhibitor PD166866 inhibits carcinogenesis and angiogenesis *in vivo* and *in vitro*

**Figure 8:**
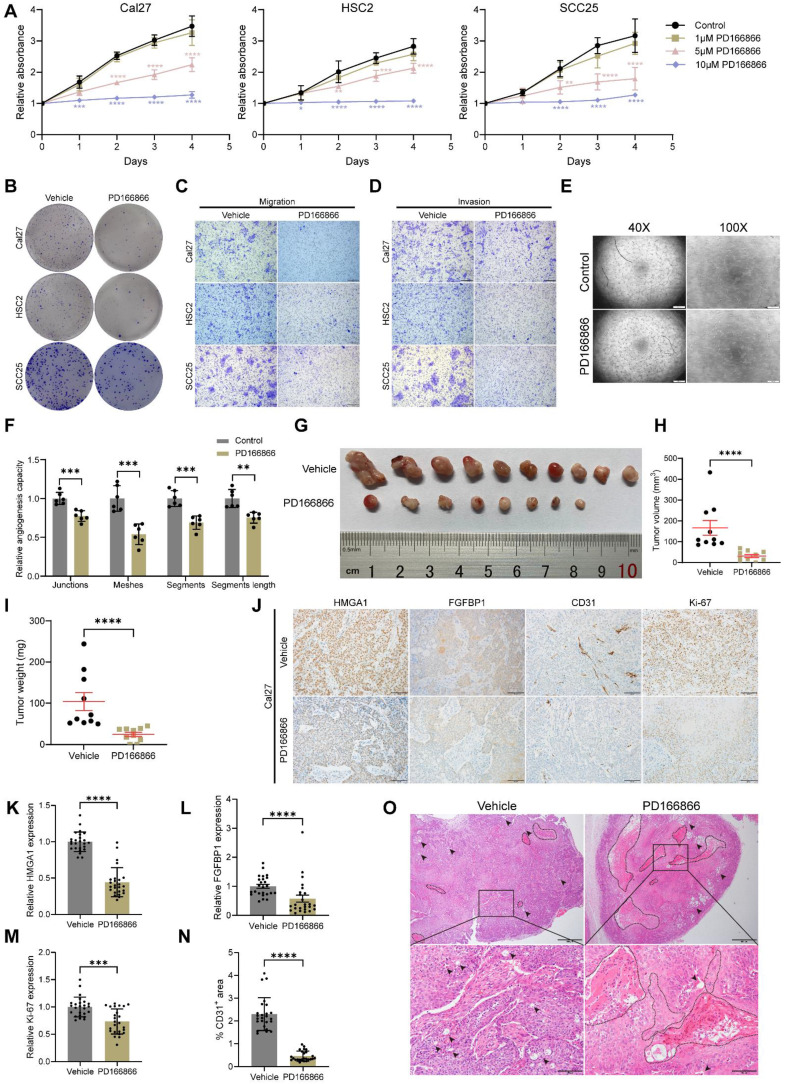
** PD166866 inhibits carcinogenesis and angiogenesis *in vivo* and *in vitro*. (A)** CCK-8 assay showing the proliferation ability of HNSCC cells with and without PD166866 treatment from 3 experiments performed in triplicate. **(B)** Clonogenic assay showing the clonogenic ability of HNSCC with and without PD166866 treatment from 3 experiments performed in triplicate. **(C)** Transwell migration assay showing the migration ability of HNSCC cells with and without PD166866 treatment following treatment with 10μM cytosine β-D-arabinoside (Ara-C) for 1 hour to mitigate effects of proliferation from 3 experiments performed in triplicate. Scale bars: 200μm. **(D)** Matrigel invasion assay showing the invasive ability of HNSCC cells with and without PD166866 treatment following treatment with 10μM Ara-C for 1 hour from 3 experiments performed in triplicate. Scale bars: 200μm. **(E, F)** Tube formation assay showing the *in vitro* angiogenic ability of HUVECs with and without PD166866 treatment from 2 experiments performed in triplicate. Scale bar, ×40 magnification, 500 μm; scale bar, ×100 magnification, 200 μm. **(G-I)** Tumor number, volume, and weight comparisons from subcutaneous implantation of Cal27 cells in mice treated with PD166866 or vehicle control (n=10/condition). **(J-N)** IHC for HMGA1, FGFBP1, CD31, Ki67 in Cal27 xenografts with PD166866 or vehicle control. Data from 5 fields at ×200 magnification of tumors from 5 different mice per condition. Scale bar, 100 μm.** (O)** Representative images of H&E staining in Cal27 xenografts with PD166866 or vehicle control. Dashed circles and arrows: cell-free and necrotic regions. Scale bar, 500 μm; scale bar, local magnification, 100μm. Data shown as mean ± SD. *P<0.05, **P<0.01, ***P<0.001, ****P<0.0001; ordinary 1-way ANOVA with Dunnett's multiple comparisons test **(A)**, 2-tailed Student's unpaired *t*-test **(F)**, Mann-Whitney test **(H, I, K, M, N)**, 2-tailed Student's unpaired *t*-test with Welch's correction**(L)**.

To improve clinical applicability, we tested PD166866, a FGFR1 inhibitor 44-46, on HNSCC. PD166866 inhibited proliferation, clonogenicity, migration, invasion of HNSCC cells, and tube formation of HUVECs **(Figs. 8A-F and S6A-E)**. In nude mice, PD166866 reduced tumor size and weight, with two tumors disappearing entirely** (Fig. 8G-I)**. IHC and IF showed lower CD31 and Ki67 levels, indicating reduced angiogenesis and proliferation **(Figs. 8J, M, N and S6F)**. PD166866 also decreased HMGA1 and FGFBP1 expression and increased necrosis in xenografts **(Fig. 8J-L, O)**.

## Discussion

Epigenetic changes, which alter gene expression and contribute to malignant transformation, are key features of tumors 15. Understanding these changes is crucial for improving cancer prevention and treatment. HMGA1, a chromatin regulator, is overexpressed in many cancers and plays a significant role in altering chromatin structure to promote malignancy 19-25. However, its function in head and neck squamous cell carcinoma (HNSCC) remains poorly understood 26-28. This study aims to explore the role of HMGA1 in HNSCC.

As a solid tumor, a troublesome feature of HNSCC is its internal blood vessels 47. Our results show that silencing* HMGA1* and its target *FGFBP1* inhibits angiogenesis, leads to tumor necrosis, and suppresses tumor progression. Additionally, silencing *HMGA1* reduces tumor stroma formation, aligning with previous studies 19. Single-cell sequencing revealed that HMGA1 is more highly expressed in malignant epithelial cells than in normal ones, indicating its role in carcinogenesis. Consistent with this, data from TCGA, GEO databases, and patient samples indicate that HMGA1 is overexpressed in HNSCC and correlates with poor prognosis. HMGA1 is known to maintain stemness in cells, and our study shows that silencing HMGA1 decreases tumor stem cells, which play a pivotal role in tumor development 48.

HMGA1 regulates gene expression, and transcriptome sequencing of HNSCC cells with and without HMGA1 silencing identified several biological processes linked to tumor progression, such as cell proliferation, migration, and angiogenesis. Notably, HMGA1 directly activates the transcription of FGFBP1, which, in turn, enhances the secretion of FGF2, a key factor in angiogenesis. Silencing FGFBP1 replicated the effects of silencing HMGA1 in both vitro and vivo experiments. Overexpression of FGFBP1 or FGF2 reversed the suppression of cell proliferation caused by HMGA1 silencing, suggesting that HMGA1 promotes tumorigenesis by upregulating FGFBP1 in HNSCC.

Further analysis showed that HMGA1-regulated genes are enriched in angiogenesis-related pathways, and previous studies have shown that HMGA1 promotes angiogenesis 25,34. In addition, FGFBP1 is the molecular chaperone of FGF2, which promotes angiogenesis by releasing FGF2, a pro-angiogenic factor. We then confirmed that HMGA1 promotes angiogenesis in HNSCC through FGFBP1 and FGF2. Silencing HMGA1 or FGFBP1 reduced FGF2 secretion and hindered angiogenesis *in vitro* and *in vivo*. Overexpression of FGFBP1 or FGF2 reversed these effects. In tumor-bearing mice, silencing HMGA1 or FGFBP1 decreased blood vessels, explaining the inhibition of tumor growth. Additionally, tumor necrosis and reduced tumor stroma formation were observed, which is consistent with the idea that silencing HMGA1 impedes angiogenesis and tumor progression.

FGFR1 inhibitor PD166866, which blocks FGF2 signaling, mimicked the effects of HMGA1 or FGFBP1 silencing, further supporting the role of the HMGA1-FGFBP1-FGF2 axis in tumor progression and angiogenesis. In HNSCC, previous studies and IHC results of our patients showed conflicting FGFBP1 expression levels 41-43. In patients with low HMGA1 expression, high FGFBP1 expression predicts a poor prognosis, and in patients with high HMGA1 expression, low FGFBP1 expression predicts a worse prognosis. This suggests that the co-survival analysis of HMGA1 and FGFBP1 can refine prognosis prediction for HNSCC patients.

Notably, although our functional experiments clearly demonstrate the direct transcriptional activation of FGFBP1 by HMGA1, their mRNA or protein levels did not show a completely consistent positive correlation in public databases or in our own clinical samples. This discrepancy may be due to the high heterogeneity of tumors, complex post-transcriptional regulation of FGFBP1, and cross-interference from other signaling pathways in the tumor microenvironment. Interestingly, the combined expression pattern of HMGA1 and FGFBP1, rather than the level of either molecule alone, exhibited stronger prognostic predictive power, suggesting that the functional relationship between the two may vary dynamically depending on the molecular context of the tumor. This further emphasizes the need to place the HMGA1-FGFBP1 axis within the broader context of the tumor ecosystem in future translational research.

IHC analysis revealed that FGFBP1 was predominantly expressed in the basal side of tumor tissues, known as the progressive frontier or tumor budding, which is associated with more malignant cells 49,50. Shahana et al. showed that the expression of FGFBP1, FGF2, and VEGFA became higher with the severity of oral epithelial dysplasia 41. We also observed higher expression of HMGA1 at this site, suggesting that HMGA1 and FGFBP1 may play a crucial role in the later stages of tumor progression. This warrants further investigation.

Anti-angiogenic therapy in HNSCC faces challenges, including low treatment efficacy and high recurrence rates 51,52. Growth factors are promising therapeutic targets, with antibodies or receptor inhibitors used to block their functions. Our study highlights the critical roles of HMGA1 and FGFBP1 in tumorigenesis and angiogenesis in HNSCC, offering new targets for anti-vascular therapy. We showed that HMGA1 upregulates FGFBP1, promoting FGF2 release, activating FGFR1, and triggering downstream pathways. This underscores the importance of the HMGA1-FGFBP1-FGF2-FGFR1 axis in HNSCC progression. Additionally, we demonstrated that targeting HMGA1 and FGFBP1 may provide a new therapeutic approach for HNSCC.

## Supplementary Material

Supplementary methods, figures and tables.

## Data Availability

The processed single-cell transcriptome data and bulk transcriptome data used in this study obtained from: Choi (GSE181919, scRNA-seq), Zhou (GSE9844, RNA-seq), Mes (GSE85446, RNA-seq), Krishnan (GSE75538, RNA-seq), Satgunaseelan (GSE184616, RNA-seq), and TCGA-HNSCC (RNA-Seq). The RNA-seq data of this study are available in the NCBI Sequence Read Archive (SRA) with the accession numbers PRJNA1178083. Data and materials included in this article are provided in the Supplementary information. Any other information is also available upon request from the authors.

## Abbreviations

Ara-C: cytosine β-D-arabinofuranoside
CCK8: cell counting kit-8
ChIP: chromatin immunoprecipitation
DEGs: differentially expressed genes
EdU: 5-ethynyl-2′-deoxyuridine
ELDA: extreme limiting dilution analysis
ELISA: enzyme-linked immunosorbent assay
FGFBP1: fibroblast growth factor-binding protein 1
FGFR1: FGF receptor 1
GO: gene ontology
H&E: hematoxylin and eosin
HMGA1: high mobility group AT-hook 1
HNSCC: head and neck squamous cell carcinoma
HOEC: human oral epithelial cells
HUVECs: human umbilical vein endothelial cells
IF: immunofluorescence
IHC: immunohistochemistry
mIF: multiple immunofluorescence
MNase: micrococcal Nuclease
OS: overall survival time
RNAseq: RNA sequencing
RT-qPCR: reverse transcriptase quantitative PCR
scRNA-seq: single-cell RNA-seq
shRNA: short hairpin RNA
ssGSEA: single sample gene set enrichment analysis
TSA: tyramide signal amplification
TSS: transcription start site
VEGF: vascular endothelial growth factor
WB: western blot
