# Supplementary material for: HMGA1 Activates FGFBP1 Transcription to Enhance Angiogenesis Induction and Tumor Progression via FGF2/FGFR1 Pathway
Source: Int J Biol Sci. 2026 Feb 26;22(6):3013–31. doi: 10.7150/ijbs.109079 (PMC13050449; doi:10.7150/ijbs.109079)
Supplement: Supplementary file 1 — Supplementary methods, figures and tables. [file ijbsv22p3013s1.pdf]

Methods

Bioinformatics analysis

scRNA-seq data were processed using "Seurat 5.0," filtering cells with 500-6000 UMI counts and excluding those with >10% mitochondrial UMIs, resulting in 53,232 cells. PCA and clustering were performed with the top 50 principal components, and nine major cell types were identified. CNVs in epithelial cells were estimated with InferCNV. DEG analysis was done using the "Limma" package (Adjusted $P < 0.05$, $|\log_2(\text{si}\textit{HMGA1}/\text{control})| \geq 0.5$), and GO enrichment was conducted using "clusterProfiler." Hallmark gene sets were analyzed via ssGSEA, and Kaplan–Meier survival analyses were performed with "survminer" and "survival."

Patient tissue samples

HNSCC tissue samples and matched adjacent non-tumorous tissues were obtained from the Department of Pathology, Shandong Provincial Hospital, collected between May 2023 and March 2024.

Cell lines and culture conditions

HNSCC cell lines (Cal27, HSC2, SCC15, SCC25), normal human oral epithelial cells (HOEC), and human umbilical vein endothelial cells (HUVECs) were used. HSC2 and HOEC were purchased from Otwo Biotech, Cal27 from Procell, SCC15 and SCC25 from ATCC, and HUVECs from Immocell. All cell lines were authenticated and mycoplasma-negative. Cells were cultured in their respective media with 10% FBS and 1% penicillin-streptomycin, under standard conditions at 37°C with 5% CO_2.

Cell transfection with siRNA, shRNA and plasmids

siRNAs and plasmids were synthesized by Genomeditech, and lentiviruses by Genechem. Transfection was performed using GMTrans Liposomal Reagent, followed by RT-qPCR and Western blotting 48 hours post-transfection. Cal27 cells were infected with lentivirus for 16 hours, then selected with puromycin (10 μg/mL) for 72 hours to generate stable clones. Silencing or overexpression efficiency was evaluated by RT-qPCR and Western blotting.

RNA isolation and reverse transcriptase quantitative PCR (RT-qPCR) assay

Total RNA was extracted using the SteadyPure Universal RNA Extraction Kit (Accurate Biology) and cDNA synthesized from 1 μg RNA using the Evo M-MLV RT Mix Kit. RT-qPCR was performed with the 2X SYBR Green Pro Taq HS Premix on the

LightCycler 480 system. Gene expression was normalized to β-actin and calculated using the $2^{-\Delta\Delta Ct}$ method.

Western blot

Cells were lysed in RIPA buffer, and protein concentration was measured using the BCA assay. Equal protein amounts were separated by SDS-PAGE, transferred to PVDF membranes, and incubated with primary and secondary antibodies. Protein bands were visualized using ECL and imaged with an Amersham Imager 600. Secreted proteins were concentrated with methanol and chloroform and analyzed by Western blotting to assess FGF2 expression.

Cell proliferation assay

Cell proliferation was assessed using the CCK-8 assay over 4 or 7 days. Cells were seeded in 96-well plates at 3,000 cells per well and incubated at 37°C with 5% CO_2. After treatments, 10 μL of CCK-8 solution was added daily, and absorbance at 450 nm was measured using a ThermoMultiskan GO. Proliferation data were normalized to the control group absorbance on day 0.

Colony formation assay

Cells were trypsinized, seeded in 6-well plates at 800-1000 cells per well, and cultured for 10-14 days at 37°C with 5% CO_2 until colonies formed. The medium was changed every 3 days. Colonies were fixed with 4% paraformaldehyde, stained with 1% crystal violet, and counted if consisting of 50 or more cells using ImageJ software.

Scratch assay

HNSCC cells were treated with 10 μM Cytosine β-D-arabinofuranoside (Ara-C) for 1 h to inhibit proliferation, and linear scratches were created in 6-well plates with 1% FBS for 20 hours. Migration was assessed by measuring scratch gaps under a microscope.

Transwell migration and Matrigel invasion assays

For migration and invasion assays, HNSCC cells were cultured in serum-free medium for 24 hours and then treated with 10 μM Ara-C for 1 hour. Cells (5×10^4) were seeded in the upper chambers of Transwell inserts, with Matrigel coating for invasion. After 20 hours of incubation, non-migrating/invading cells were removed, and those on the lower surface were fixed, stained with crystal violet, washed, and photographed.

In vivo limiting dilution assay

To evaluate tumor-initiating potential, varying cell numbers (1×10^4, 5×10^4, and 1×10^5) were injected into BALB/c nude mice and monitored for 5, 6, and 9 weeks, respectively. Tumor volume was calculated using the formula: volume = 0.5 × length × width² [35]. Tumor-initiating cell frequency was assessed by Extreme Limiting Dilution Analysis (ELDA), with statistical significance determined using the chi-square test [36]. Mice were kept in SPF conditions.

RNA sequencing (RNAseq)

RNA was extracted using RNAex Pro Reagent, and library construction and sequencing were performed by the Beijing Genomics Institute. Data analysis was done using an internal assembler and variant caller. Clean reads were aligned to the reference genome with STAR, and gene read counts were obtained with HTSeq. Differential expression was analyzed using the edgeR package, with genes showing a $\log_2$ fold change ≥ 0.5 and adjusted $P < 0.05$ considered significantly different (Supplemental Table 1).

Ethynyl-2-deoxyuridine (EdU) proliferation assay

Cell proliferation in Cal27 cells was assessed using the EdU Apollo DNA Kit. Transfected cells and controls (3×10^3 cells/well) were seeded in 96-well plates and treated with human FGF2 protein. After 72 hours, cells were incubated with 20 μM EdU for 2 hours, fixed, and stained with Apollo 488 for EdU detection and Hoechst 33342 for nuclear staining. EdU-positive cells were quantified using ImageJ software.

Chromatin immunoprecipitation (ChIP)-quantitative PCR (qPCR) assay

ChIP assays were performed using the Pierce™ Agarose ChIP Kit. Chromatin was cross-linked, sheared, and treated with Micrococcal Nuclease. The FGFBP1 promoter region, predicted to contain an HMGA1 binding site, was amplified using primers designed for a 142 bp product (Supplementary Table 1). After immunoprecipitation, DNA was purified and analyzed by qPCR, with specificity confirmed by gel electrophoresis. Enrichment of the FGFBP1 promoter was calculated relative to a nonspecific IgG control and normalized to input DNA.

Agarose gel electrophoresis

Agarose gel electrophoresis was performed to assess DNA fragmentation and PCR product specificity. A 1.2% agarose gel was

prepared with TAE buffer, stained with nucleic acid dye, and loaded with DNA samples mixed with loading buffer. DNA fragments were sized using a DNA ladder and electrophoresed at 120 V. Bands were visualized using a UV transilluminator.

Dual-luciferase reporter assay

The dual-luciferase reporter assay was used to assess HMGA1's transcriptional activation of the FGFBP1 promoter. Cal27 cells were co-transfected with firefly luciferase plasmids (wild-type and mutated FGFBP1 promoter regions) and a Renilla luciferase control plasmid. After 48 hours, cell lysates were prepared, and luciferase activities were measured using the Dual-Luciferase Reporter Assay Kit. Firefly luciferase activity was normalized to Renilla activity, and relative luciferase activity was calculated as the ratio of firefly to Renilla luminescence.

Enzyme-linked immunosorbent assay (ELISA) for human FGF2

FGF2 secretion by Cal27 cells was measured using the Human FGF2 ELISA Kit. Conditioned media or standards (100 μL) were added to a pre-coated 96-well plate and incubated for 2 hours at 37°C. After washing, biotinylated detection antibody and HRP-streptavidin were added. Following substrate addition, absorbance at 450 nm was measured, and FGF2 concentrations were determined from a standard curve and expressed in pg/mL.

Preparation of conditioned medium

Conditioned medium for ELISA, Western blot, CCK-8, tube formation, and Matrigel plug assays was prepared by culturing Cal27 cells in serum-free DMEM for 24 hours after reaching 80% confluence. The medium was collected, centrifuged at 3,000 rpm for 20 minutes, and stored at -80°C for downstream analysis to assess its effects on HUVECs.

Transwell co-culture migration assays

Transwell co-culture migration assays were performed in 24-well plates with 8 μm pore size. Cal27 cells were plated in the bottom chamber, and HUVECs (3×10^4 cells/well) were seeded in the upper chamber in serum-free ECM. After 20 hours of incubation at 37°C with 5% CO_2, migrated HUVECs were stained and photographed.

Tube formation assay

Matrigel was mixed with Cal27 supernatants in a 1:1 ratio and added to 96-well plates. After 30 minutes, HUVECs (2.5×10^4

cells/well) were seeded and incubated for 1-3 hours. For PD166866 treatment, Matrigel was mixed with DMEM, and HUVECs were treated with PD166866 for 24 hours before seeding in ECM. High-resolution images of the capillary-like structures were captured using an inverted light microscope. Quantitative analysis was performed using the Angiogenesis Analyzer plugin for ImageJ software (NIH). To comprehensively assess the angiogenic ability, the following four parameters were quantified: 1) Number of junctions: the number of branching points connecting segments, indicating the complexity of the vascular network; 2) Number of meshes: the number of enclosed polygonal areas, reflecting the maturity of tube formation; 3) Number of segments: the count of individual linear vessel elements delimited by junctions; 4) Total segments length: the sum of the lengths of all segments, representing the overall extension capability of the endothelial cells.

Matrigel plug assay

A Matrigel plug assay was performed to assess the effects of Cal27 supernatants on HUVECs. A mixture of 100 μL Matrigel, 100 μL Cal27 supernatants, and 1×10^6 HUVECs was implanted into nude mice. After two weeks, the mice were euthanized, and the Matrigel plugs were excised for analysis.

Hematoxylin and eosin (H&E), immunohistochemistry (IHC) and immunofluorescence (IF)

Tissue samples were fixed in 4% paraformaldehyde, dehydrated, cleared in xylene, and embedded in paraffin. Sections (5 μm) were deparaffinized, rehydrated, and stained with hematoxylin and eosin (H&E) for light microscopy imaging. For IHC, sections underwent antigen retrieval, blocking, and incubation with primary and secondary antibodies. Immunoreactivity was detected using a DAB Substrate Kit, and nuclei were counterstained with hematoxylin. Staining intensity, area, and number were quantified using ImageJ software. For immunofluorescence (IF), sections followed the same antigen retrieval and blocking steps, incubated with primary antibodies, and then fluorophore-conjugated secondary antibodies. Nuclei were counterstained with DAPI, and images were captured using a digital slice scanner.

Multiple immunofluorescence (mIF)

For mIF, 5 μm tissue sections were deparaffinized, rehydrated, and subjected to antigen retrieval in sodium citrate buffer. After blocking with 3% BSA, sections were incubated with primary antibodies (CD31, HMGA1, FGFBP1) overnight at 4°C, followed by

secondary antibody incubation and fluorescein-conjugated tyramide signal amplification (TSA). Microwave treatment was applied to remove antibodies, and blocking was repeated. The last primary antibody (anti-FGF2) was incubated overnight, followed by Cy3-conjugated secondary antibody. Nuclei were stained with DAPI, and images were scanned using a digital slice scanner.

Masson's trichrome staining

Tissue sections were deparaffinized by baking at 65°C for 60 minutes, followed by immersion in xylene and rehydration through graded ethanol. Slides were mordanted with Bouin's solution, stained with Weigert's hematoxylin, and differentiated in acidic ethanol. Sections were stained with Biebrich scarlet-acid fuchsin, treated with phosphomolybdic acid, and stained with aniline blue. After dehydration, clearing in xylene, and mounting with neutral balsam, images were scanned using a digital slice scanner.

Tumor-bearing mouse model and PD166866 treatment

To establish a tumor-bearing mouse model, 8×10^5 Cal27 cells were injected subcutaneously into BALB/c nude mice. When tumors reached 20-30 mm^3, mice were randomized into two groups: PD166866 treatment (30 mg/kg every two days for 30 days) or vehicle control (0.5% sodium carboxymethyl cellulose in saline).

Statistical analysis

Statistical analyses were performed using GraphPad Prism 9.0. Normality was assessed with the Shapiro-Wilk test, and variances were evaluated using the F test or Bartlett test. For two-group comparisons, a 2-tailed Student's unpaired t-test was used for normally distributed data with equal variances, Welch's correction for unequal variances, and the Mann-Whitney test for non-normally distributed data. For multiple groups, one-way ANOVA with Dunnett's multiple comparisons test was applied for normally distributed data, and the Kruskal-Wallis test for non-normally distributed data. Two-way ANOVA with Šídák's test was used for two-factor comparisons. Survival was analyzed by the log-rank test, and correlation was assessed using Pearson's correlation coefficients. A p-value < 0.05 was considered statistically significant. Data are presented as mean ± SD or SEM.

Figure S1. HMGA1 is highly expressed and is associated with poor prognosis in HNSCC. (A) The HMGA1 expression profile across all tumor samples and paired normal tissues. Data was downloaded from GEPIA database. **(B)** Copy number variations (CNVs) evaluated epithelial cells by InferCNV, macrophages cells were used as control group. **(C)** Boxplot showing expression of HMGA1 between normal (n=12 patients) and tumor (n=26 patients) in the GSE9844 cohort. **(D)** Boxplot showing expression of HMGA1 between normal (n=14 patients) and tumor (n=14 patients) in the GSE75538 cohort. **(E)** Boxplot plot showing expression of HMGA1 between normal (n=15 patients) and tumor (n=15 patients) in the GSE184616 cohort. **(F)** The Kaplan-Meier overall survival curves between low-HMGA1 group (n=16 patients) and high-HMGA1 group (n=50 patients) of GSE85446 cohort. Results are shown as mean ± standard deviation (SD). *$P<0.05$, **$P<0.01$; 2-tailed Student's unpaired *t*-test **(C-E)**, log-rank test **(F)**.

Figure S2. HMGA1 is highly expressed in HNSCC cell lines and its silencing disrupts oncogenic properties. (A) qRT-PCR showing the expression of HMGA1 mRNA in human oral epithelial cells (HOEC) and HNSCC cell lines (Cal27, HSC2, SCC25, SCC15) from 1 experiment performed in triplicate. **(B)** Western blot (n = 3 experiments) showing the expression of HMGA1 protein in HNSCC cell lines. **(C, D)** Scratch assay showing the migration ability of HNSCC cells with and without *HMGA1* silencing following treatment with 10μM cytosine β-D-arabinoside (Ara-C) for 1 hour to mitigate effects of proliferation from 3 experiments performed in triplicate. Scale bars: 500μm. **(E)** Quantification of Transwell migration assay showing the migration ability of HNSCC cells with and without *HMGA1* silencing following treatment with 10μM Ara-C for 1 hour from 3 experiments performed in triplicate. **(F, G)** In vivo limiting dilution assay showing xenograft tumorigenicity of Cal27 cells with and without *HMGA1* silencing at different limiting dilutions (n=10/condition). Data shown as mean ± SD **(A, D, E)** or mean ± standard error of the mean (SEM) **(F, G)**. *$P<0.05$, **$P<0.01$, ***$P<0.001$, ****$P<0.0001$; 2-tailed Student's unpaired *t*-test with Welch's correction (Cal27 of **A**), 2-tailed Student's unpaired *t*-test (HSC2, SCC25 and SCC15 of **A),** Brown-Forsythe and Welch's ANOVA test with Dunnett's T3 multiple-comparisons test (Cal27 of **D**, HSC2 of **E**), Kruskal-Wallis test with Dunn's multiple-comparison test (HSC2 of **D**, Cal27 of **E**), ordinary 1-way ANOVA with Dunnett's multiple comparisons test (SCC25 of **D,** SCC25 of **E**), Mann-Whitney test **(F, G)**.

Figure S3. Overexpression of FGFBP1 rescues the proliferation inhibition caused by *HMGA1* silencing. (A, B) Scratch assay showing the migration ability of HNSCC cells with and without *HMGA1* silencing following treatment with 10μM cytosine β-D-arabinoside (Ara-C) for 1 hour to mitigate effects of proliferation from 3 experiments performed in triplicate. Scale bars: 500μm. **(C)** Quantification of Transwell migration assay showing the migration ability of HNSCC cells with and without *HMGA1* silencing following treatment with 10μM Ara-C for 1 hour from 3 experiments performed in triplicate. **(D, E)** qRT-PCR and western blot (n = 3 independently biological experiments in **D** and **E**) showing the expression of FGFBP1 mRNA and protein in Cal27 cells with and without FGFBP1 overexpression via plasmid delivery. **(F, G)** EdU (5-ethynyl-2'-deoxyuridine) incorporation assay showing proliferation of Cal27 cells under different conditions (control, *HMGA1* silencing, *FGFBP1* silencing, *HMGA1* silencing + *FGFBP1* overexpressing, *HMGA1* silencing + 100ng/mL hFGF2). Data from 3 fields per sample from 2 experiments performed in triplicate. Scale bar, 100 μm. **(H, I)** In vivo limiting dilution assay showing xenograft tumorigenicity of Cal27 cells with and without *FGFBP1* silencing at different limiting dilutions (n=10/condition). Data shown as mean ± SD **(B-D, G)** or mean ± SEM **(H, I)**. *$P<0.05$, **$P<0.01$, ***$P<0.001$, ****$P<0.0001$; ordinary 1-way ANOVA with Dunnett's multiple comparisons test **(Cal27 and HSC2 of B)**, Brown-Forsythe and Welch's ANOVA test with Dunnett's T3 multiple-comparisons test **(SCC25 of B, HSC2 of C)**, Kruskal-Wallis test with Dunn's multiple-comparison test **(Cal27, SCC25 of C, G)**, 2-tailed Student's unpaired t-test with Welch's correction **(D)**, Mann-Whitney test **(H, I)**.

Figure S4. HMGA1 and FGFBP1 promote angiogenesis in vivo. (A) Immunofluorescence (IF) for CD31 of the Matrigel plug showing the in vivo angiogenic ability of HUVECs treated with different conditioned supernatants of Cal27 cells. Scale bar, 500 μm; scale bar, local magnification, 50 μm. **(B)** IF for CD31 showing the in vivo angiogenic ability of Cal27 xenografts with and without *HMGA1* or *FGFBP1* silencing. Scale bar, 500 μm; scale bar, local magnification, 100μm.

Figure S5. HMGA1 and FGFBP1 together predict the prognosis of HNSCC patients. (A) The FGFBP1 expression profile across all tumor samples and paired normal tissues (GEPIA). **(B)** Boxplot showing expression of FGFBP1 between normal (n=44

patients) and tumor (n=516 patients) in the TCGA-HNSCC cohort. **(C)** Boxplot showing expression of FGFBP1 between normal (n=12 patients) and tumor (n=26 patients) in the GSE9844 cohort. **(D)** Boxplot showing expression of FGFBP1 between normal (n=14 patients) and tumor (n=14 patients) in the GSE75538 cohort. **(E)** Boxplot plot showing expression of FGFBP1 between Normal (n=15 patients) and Tumor (n=15 patients) in the GSE184616 cohort. **(F, G)** IHC showing the expression of FGFBP1 in adjacent tissues (n=13 patients) and tumors (n=29 patients) with HNSCC. **(H)** The Kaplan-Meier overall survival curves between low- FGFBP1 group (n=177 patients) and high- FGFBP1 group (n=339 patients) of TCGA-HNSCC cohort. **(I)** Kaplan-Meier survival curves showing high HMGA1 and FGFBP1 expression (light blue, n =162 patients), high HMGA1 and low FGFBP1 expression (red, n=177 patients), low HMGA1 and high FGFBP1 expression (dark blue, n=75 patients), and low HMGA1 and FGFBP1 expression (black, n=102 patients) in the TCGA-HNSCC cohort. Patients are categorized into high- and low- groups based on the optimal cutoff value of gene expression level in **(I)**. Results are shown as mean ± standard deviation (SD). ns, not significant; **P<0.01, ***P<0.001; 2-tailed Student's unpaired t-test **(B, C, D, E)**, Mann-Whitney test **(F)**, log-rank test **(H, I)**.

Figure S6. PD166866 suppresses HNSCC cell malignancy and angiogenesis in vivo. (A) Quantification of clonogenic assay showing the migration ability of HNSCC cells with and without PD166866 addition from 3 experiments performed in triplicate. **(B)** Quantification of Transwell migration assay showing the migration ability of HNSCC cells with and without PD166866 addition following treatment with 10μM Ara-C for 1 hour from 3 experiments performed in triplicate. **(C, D)** Scratch assay showing the migration ability of HNSCC cells with and without PD166866 addition following treatment with 10μM cytosine β-D-arabinoside (Ara-C) for 1 hour to mitigate effects of proliferation from 3 experiments performed in triplicate. Scale bars: 500μm. **(E)** Quantification of Matrigel invasion assay showing the invasive ability of HNSCC cells with and without PD166866 addition following treatment with 10μM Ara-C for 1 hour from 3 experiments performed in triplicate. **(F)** IF for CD31 showing the in vivo angiogenic ability of Cal27 xenografts with and without PD166866 treatment. Scale bar, 500 μm; scale bar, local magnification, 100μm. Results are shown as mean ± SD. *P<0.05, **P<0.01, ****P<0.0001; 2-tailed Student's unpaired t-test (A, Cal27 and SCC25 of B, D, Cal27 and HSC2 of E), 2-tailed Student's unpaired t-test with Welch’s correction (HSC2 of B), Mann-Whitney test (SCC25 of E).

Figure S1

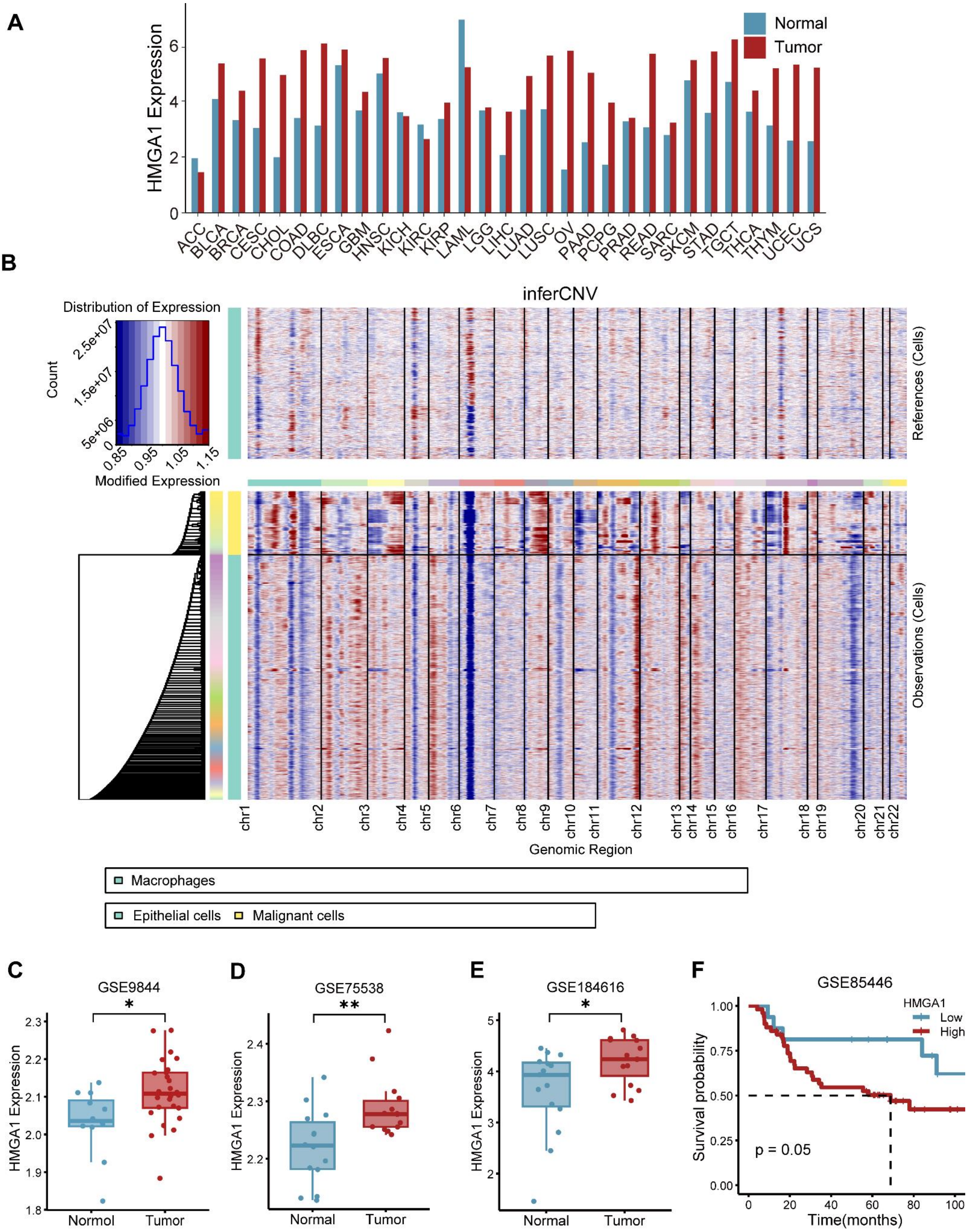
A
HMGA1 Expression
Normal
Tumor
ACC BLCA BRCA CESC CHOL COAD DLBC ESCA GBM HNSC KICH KIRC KIRP LAML LGG LIHC LUAD LUSC OV PAAD PCPG PRAD READ SARC SKCM STAD TGCT THCA THYM UCEC UCS
B
inferCNV
Distribution of Expression
Count
Modified Expression
References (Cells)
Observations (Cells)
chr1 chr2 chr3 chr4 chr5 chr6 chr7 chr8 chr9 chr10 chr11 chr12 chr13 chr14 chr15 chr16 chr17 chr18 chr19 chr20 chr21 chr22
Genomic Region
Macrophages
Epithelial cells
Malignant cells
C
GSE9844
HMGA1 Expression
Normol
Tumor
D
GSE75538
HMGA1 Expression
Normal
Tumor
E
GSE184616
HMGA1 Expression
Normal
Tumor
F
GSE85446
HMGA1
Low
High
Survival probability
p = 0.05
Time(months)

Figure S2

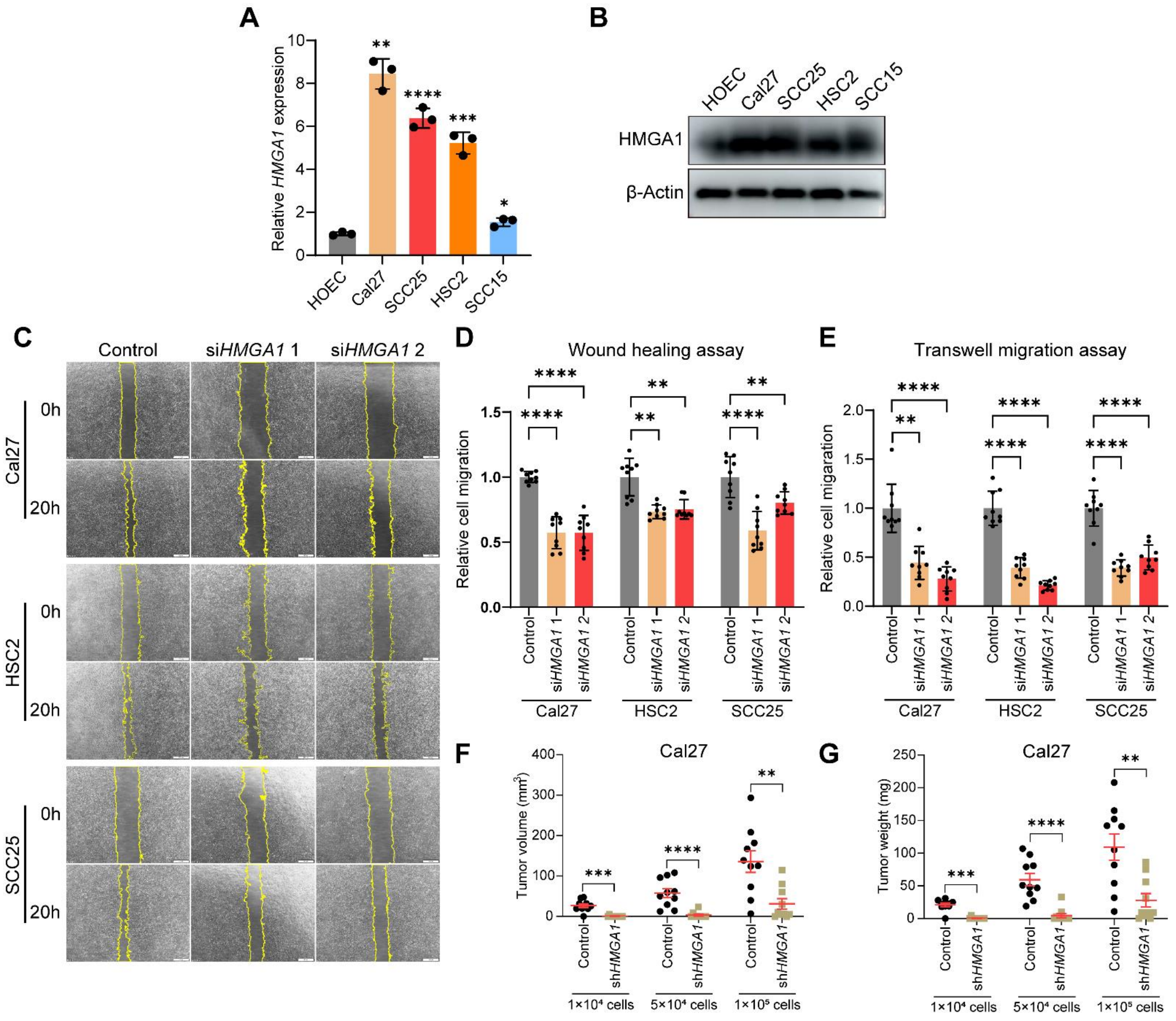
A
B
C
D
E
F
G
Relative HMGA1 expression
HOEC
Cal27
SCC25
HSC2
SCC15
HMGA1
β-Actin
Control
siHMGA1 1
siHMGA1 2
0h
20h
Wound healing assay
Relative cell migration
Transwell migration assay
Relative cell migaration
Tumor volume (mm³)
Tumor weight (mg)
shHMGA1
1×10⁴ cells
5×10⁴ cells
1×10⁵ cells

Figure S3

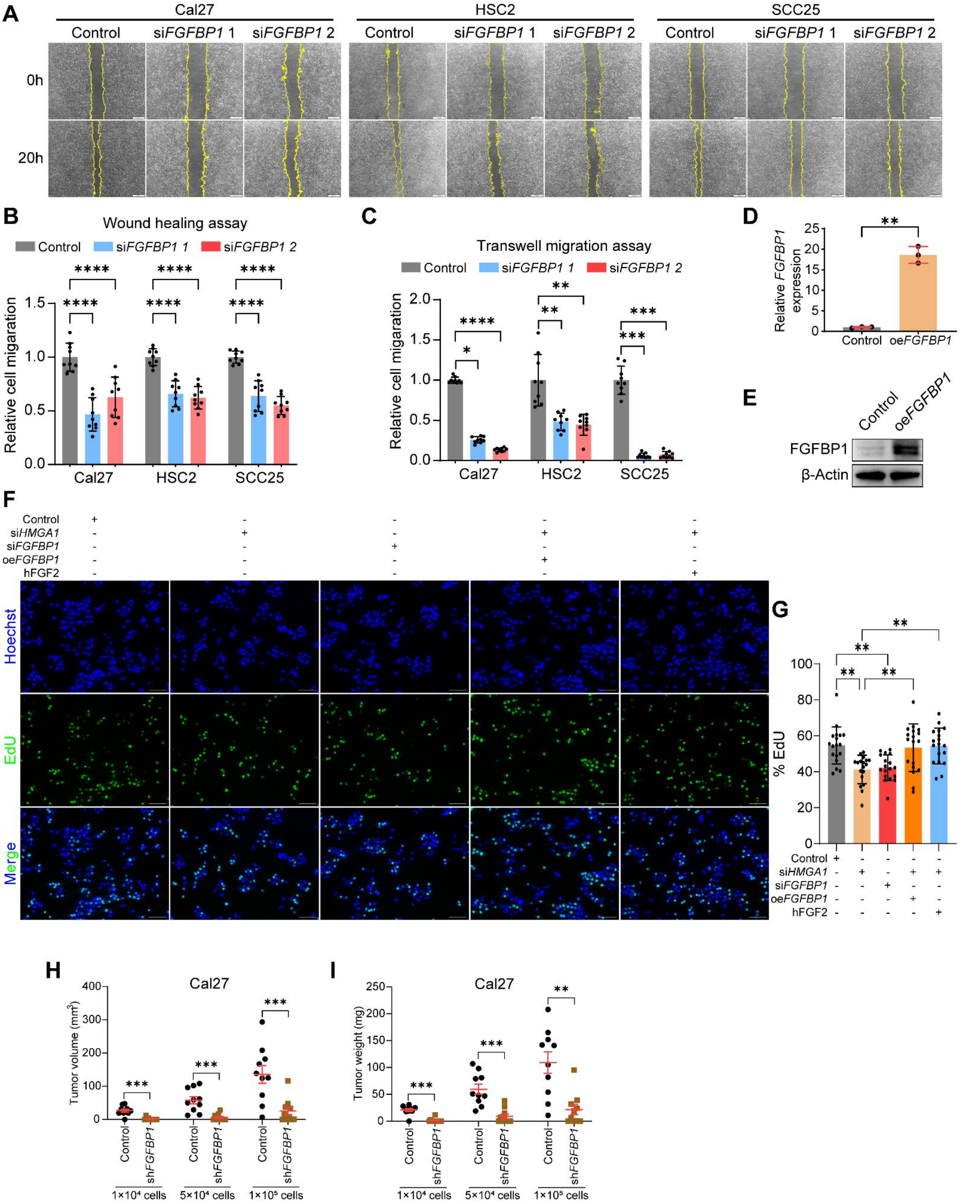
A
Cal27
HSC2
SCC25
Control
siFGFBP1 1
siFGFBP1 2
0h
20h
B
Wound healing assay
Control
siFGFBP1 1
siFGFBP1 2
Relative cell migaration
Cal27
HSC2
SCC25
C
Transwell migration assay
Control
siFGFBP1 1
siFGFBP1 2
Relative cell migaration
Cal27
HSC2
SCC25
D
Relative FGFBP1 expression
Control
oeFGFBP1
E
Control
oeFGFBP1
FGFBP1
β-Actin
F
Control
siHMGA1
siFGFBP1
oeFGFBP1
hFGF2
Hoechst
EdU
Merge
G
% EdU
Control
siHMGA1
siFGFBP1
oeFGFBP1
hFGF2
H
Cal27
Tumor volume (mm³)
Control
shFGFBP1
1×10⁴ cells
5×10⁴ cells
1×10⁵ cells
I
Cal27
Tumor weight (mg)
Control
shFGFBP1
1×10⁴ cells
5×10⁴ cells
1×10⁵ cells

Figure S4

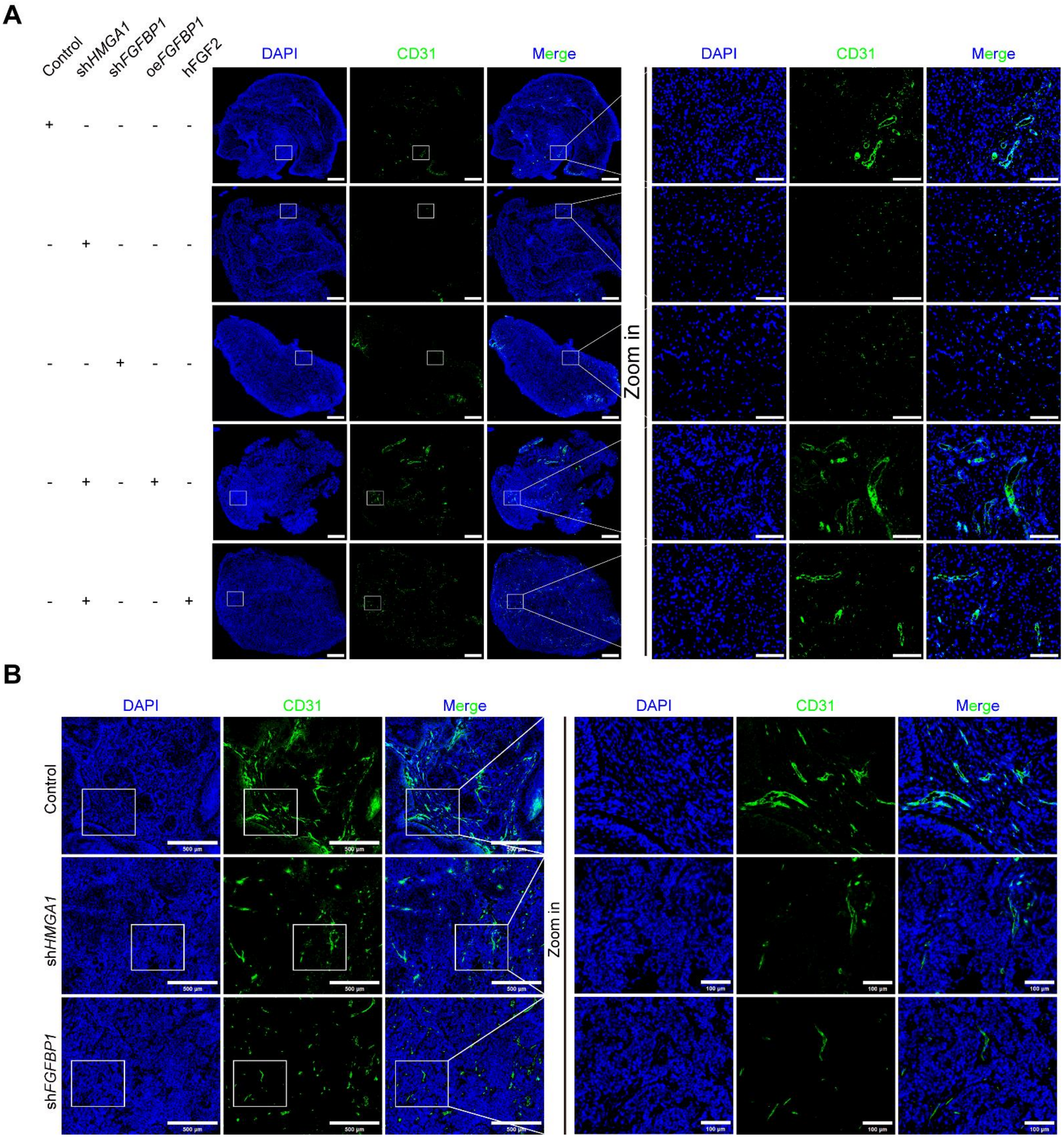
A
Control
shHMGA1
shFGFBP1
oeFGFBP1
hFGF2
DAPI
CD31
Merge
Zoom in
B
Control
shHMGA1
shFGFBP1
500 μm
100 μm

Figure S5

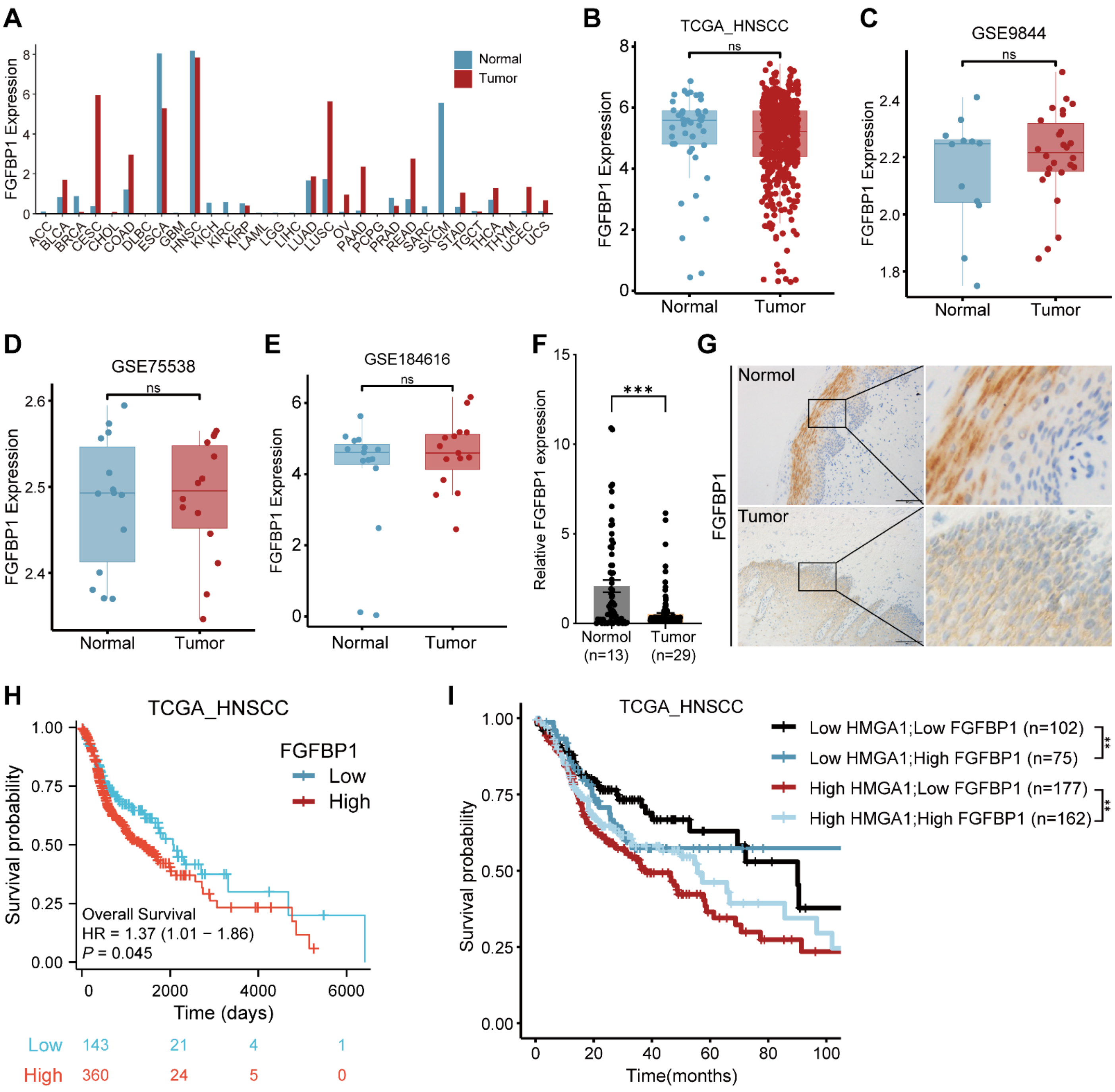
A
FGFBP1 Expression
Normal
Tumor
ACC
BLCA
BRCA
CESC
CHOL
COAD
DLBC
ESCA
GBM
HNSC
KICH
KIRC
KIRP
LAML
LGG
LIHC
LUAD
LUSC
OV
PAAD
PCPG
PRAD
READ
SARC
SKCM
STAD
TGCT
THCA
THYM
UCEC
UCS
B
TCGA_HNSCC
ns
FGFBP1 Expression
Normal
Tumor
C
GSE9844
ns
FGFBP1 Expression
Normal
Tumor
D
GSE75538
ns
FGFBP1 Expression
Normal
Tumor
E
GSE184616
ns
FGFBP1 Expression
Normal
Tumor
F
Relative FGFBP1 expression
***
Normol (n=13)
Tumor (n=29)
G
FGFBP1
Normol
Tumor
H
TCGA_HNSCC
FGFBP1
Low
High
Survival probability
Overall Survival
HR = 1.37 (1.01 − 1.86)
P = 0.045
Time (days)
Low 143 21 4 1
High 360 24 5 0
I
TCGA_HNSCC
Low HMGA1;Low FGFBP1 (n=102)
Low HMGA1;High FGFBP1 (n=75)
High HMGA1;Low FGFBP1 (n=177)
High HMGA1;High FGFBP1 (n=162)
**
**
Survival probability
Time(months)

Figure S6

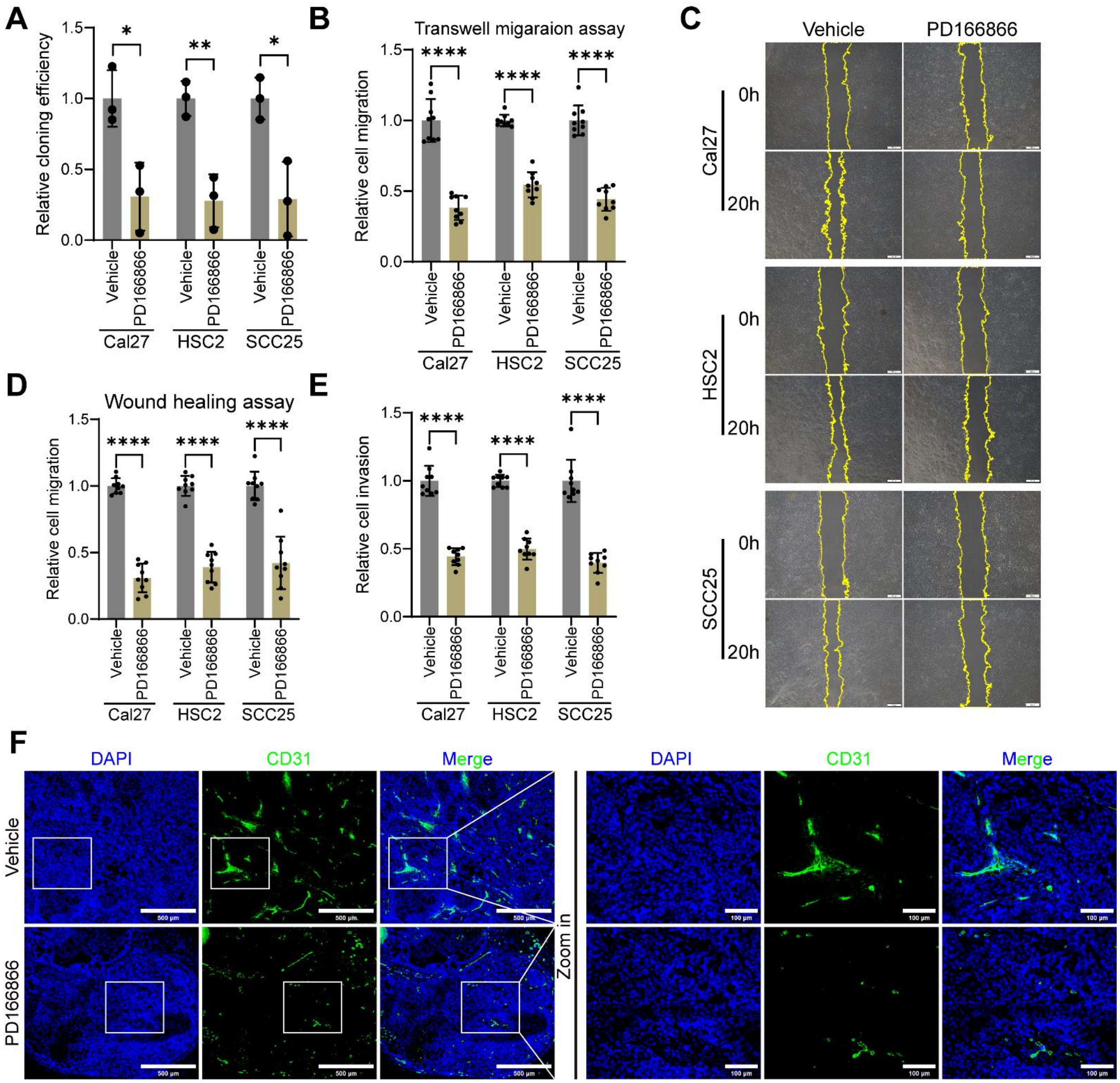
A
B
C
D
E
F
Relative cloning efficiency
1.5
1.0
0.5
0.0
*
**
Vehicle
PD166866
Cal27
HSC2
SCC25
Transwell migaraion assay
Relative cell migration
****
Vehicle
PD166866
0h
20h
Wound healing assay
Relative cell invasion
DAPI
CD31
Merge
Zoom in
500 μm
100 μm

Supplemental Table 1. Sequence information for primers, siRNAs, and shRNAs.

**Primer name**	**Species**	**Direction**	**Sequence (5'-3')**	**Application**
β-Actin	Human	Forward	TGGCACCCAGCACAATGAA	qPCR
		Reverse	CTAAGTCATAGTCCGCCTAGAAGCA	qPCR
HMGA1	Human	Forward	AGCGAAGTGCCAACACCTAAG	qPCR
		Reverse	CTCCTCTTCCTCCTTCTCCAGTT	qPCR
FGFBP1	Human	Forward	CCTGCTCTCCTTCCTCCTACT	qPCR
		Reverse	GTGTTGCCCAGAGTGTCCTTT	qPCR
HMGA1-siRNA #1	Human	Forward	CACAUGCCCUCCUGGACAAtt	Gene silencing
		Reverse	UUGUCCAGGAGGGCAUGUGtt	Gene silencing
HMGA1-siRNA #2	Human	Forward	CUCACCACCACACUACACAdTdT	Gene silencing
		Reverse	UGUGUAGUGUGGUGGUGAGdTdT	Gene silencing
HMGA1-siRNA #3	Human	Forward	GAGUACAUAUUGUGGUGAUdTdT	Gene silencing
		Reverse	AUCACCACAAUAUGUACUCdTdT	Gene silencing
FGFBP1-siRNA #1	Human	Forward	GCCUAAAGCUCAAGGAUGATT	Gene silencing
		Reverse	UCAUCCUUGAGCUUUAGGCTT	Gene silencing
FGFBP1-siRNA #2	Human	Forward	GCAAAGUGGUCUCAGAACATT	Gene silencing
		Reverse	UGUUCUGAGACCACUUUGCTT	Gene silencing
shHMGA1	Human	Forward	CcggGAGGGCATCTCTCTCAAGGTTTTCAAGAGAAACCTTGAGAGAGATGCCCTCTTTTTg	Gene silencing
		Reverse	aattcaaaaaGAGGGCATCTCTCTCAAGGTTTCTCTTGAAAACCTTGAGAGAGATGCCCTC	Gene silencing
shFGFBP1	Human	Forward	CcggCACATGCCCTCCTGGACAACTCGAGTTGTCCAGGAGGGCATGTGTTTTTg	Gene silencing
		Reverse	aattcAAAAACACATGCCCTCCTGGACAACTCGAGTTGTCCAGGAGGGCATGTG	Gene silencing
FGFBP1	Human	Forward	GTGTGACAATACTGAGCCTAATCC	ChIP-qPCR
		Reverse	GCAACTGACTTTTGGGTTTGAGAT	ChIP-qPCR

Supplemental Table 2. Antibodies for WB, IHC, IF, mIF and ChIP, and TSA for mIF.

Primary Antibody	Cat No.	Company	Dilution	Application
Anti-HMGA1	ab129153	Abcam	1:3000	Western blot
Anti-GAPDH	10494-1-AP	Proteintech	1:5000	Western blot
Anti-FGFBP1	MAB1593	R&D Systems	1 μg/mL	Western blot
Anti-FGF2	ab208687	Abcam	1:1000	Western blot
Anti-pFGFR1	AP0036	Abclonal	1:1000	Western blot
Anti-FGFR1	CY5610	Abways	1:1000	Western blot
Anti-pERK	CY5277	Abways	1:1000	Western blot
Anti-ERK	CY5487	Abways	1:1000	Western blot
Anti-pAKT	CY6569	Abways	1:1000	Western blot
Anti-AKT	60203-2-lg	Proteintech	1:5000	Western blot
Anti-β-Actin	20536-1-AP	Proteintech	1:4000	Western blot
Anti-HMGA1	ab129153	Abcam	1：1000	IHC for tumors of mice
Anti-FGFBP1	25006-1-AP	Proteintech	1：400	IHC for tumors of mice
Anti-CD31	28083-1-AP	Proteintech	1：2000	IHC for tumors of mice
Anti-Ki67	28074-1-AP	Proteintech	1：1000	IHC for tumors of mice
Anti-HMGA1	ab129153	Abcam	1：1000	IHC for tissues of patients
Anti-FGFBP1	25006-1-AP	Proteintech	1：200	IHC for tissues of patients
Anti-CD31	28083-1-AP	Proteintech	1：1000	IF for tumors of mice
Anti-CD31	28083-1-AP	Proteintech	1：4000	mIF
Anti-HMGA1	ab129153	Abcam	1：500	mIF
Anti-FGFBP1	25006-1-AP	Proteintech	1：1000	mIF
Anti-FGF2	ab208687	Abcam	1：1000	mIF

Antibody	Cat No.	Company	Amount (μg)	Application
Anti-HMGA1	ab129153	Abcam	2μg	ChIP
Anti-HMGA1	ab252930	Abcam	2μg	ChIP
Rabbit IgG	26156	Thermo	2μg	ChIP

Secondary Antibody	Cat No.	Company	Dilution	Application
HRP-conjugated Goat Anti-Rabbit IgG(H+L)	SA00001-2	Proteintech	1:5000	Western blot
HRP-conjugated Goat Anti-Mouse IgG(H+L)	SA00001-1	Proteintech	1:5000	Western blot
Goat Anti - Rabbit IgG (H+L) HRP	EF0002	SparkJade	1 : 200	IHC
Goat Anti-Rabbit IgG (H+L) - Alexa Fluor 488	EF0008	SparkJade	1:200	IF
HRP-conjugated Goat Anti-Rabbit IgG (H+L)	GB23303	Servicebio	1:500	mIF for anti-CD31, FGF2, FGFBP1
Cy3-conjugated Goat Anti-Rabbit IgG (H+L)	GB21303	Servicebio	1:300	mIF for anti-HMGA1

Fluorescein-conjugated TSA	Cat No.	Company	Dilution	Application
iF488-Tyramide	G1231	Servicebio	1:500	mIF for CD31
iF647-Tyramide	G1232	Servicebio	1:500	mIF for FGF2
iF440-Tyramide	G1250	Servicebio	1:500	mIF for FGFBP1

Supplemental Table 3. DEGs in Cal27 with and without *HMGA1* silencing (adjusted $P < 0.05$, $|\log_2(\text{si}HMGA1/\text{control})| \geq 0.5$).

Gene ID	Gene Symbol	Type	$\log_2$(si*HMGA1* / Control)	$-\log_{10}$ (adjusted *P* value)
922	'CD5L'	mRNA	-4.584938797	1.561729652
3159	'HMGA1'	mRNA	-2.770144526	207.1764648
267004	'PGBD3'	mRNA	-2.386396202	1.687163848
414325	'DEFB103A'	mRNA	-1.99880389	5.694969072
5055	'SERPINB2'	mRNA	-1.962919434	11.49431126
79047	'KCTD15'	mRNA	-1.834166968	2.484789938
3853	'KRT6A'	mRNA	-1.591257147	26.74308117
114801	'TMEM200A'	mRNA	-1.394178417	2.565958317
9982	'FGFBP1'	mRNA	-1.373323659	58.83922007
84659	'RNASE7'	mRNA	-1.337563359	100.5040413
8728	'ADAM19'	mRNA	-1.225852349	5.301039881
27063	'ANKRD1'	mRNA	-1.219107014	118.0798828
80115	'BAIAP2L2'	mRNA	-1.20874438	2.654234272
1848	'DUSP6'	mRNA	-1.157861579	25.45886218
3572	'IL6ST'	mRNA	-1.157290362	177.4402592
389161	'ANKUB1'	mRNA	-1.111133718	1.976997204
53344	'CHIC1'	mRNA	-1.100155242	16.0781905
9308	'CD83'	mRNA	-1.085711133	31.65869461
55884	'WSB2'	mRNA	-1.070136576	56.09555039
23670	'CEMIP2'	mRNA	-1.062638163	93.08885123
80183	'RUBCNL'	mRNA	-1.052056824	4.15634437
51561	'IL23A'	mRNA	-1.020601301	7.267125324
54626	'HES2'	mRNA	-1.01404957	1.564593943
3673	'ITGA2'	mRNA	-1.005483324	56.58601142
80352	'RNF39'	mRNA	-1.0002171	2.274739472
5445	'PON2'	mRNA	-0.963464072	42.25741972
219902	'TLCD5'	mRNA	-0.957718941	1.506855782
114548	'NLRP3'	mRNA	-0.952278795	1.380157791
51257	'MARCHF2'	mRNA	-0.943870195	3.200623398
3908	'LAMA2'	mRNA	-0.911218612	1.31010287
1490	'CCN2'	mRNA	-0.909727989	13.64301262
22934	'RPIA'	mRNA	-0.905699091	30.3134332
121268	'RHEBL1'	mRNA	-0.898876893	2.297106761
51129	'ANGPTL4'	mRNA	-0.88846102	6.464522239
1847	'DUSP5'	mRNA	-0.884244448	45.58456495
894	'CCND2'	mRNA	-0.877183591	54.67897859
1026	'CDKN1A'	mRNA	-0.866364146	21.4769562
5099	'PCDH7'	mRNA	-0.862354839	1.453841383
58504	'ARHGAP22'	mRNA	-0.855524192	1.498493877
3918	'LAMC2'	mRNA	-0.848822297	24.60615517
10974	'ADIRF'	mRNA	-0.843734736	5.101569533
54739	'XAF1'	mRNA	-0.841143611	5.301685865
388650	'DIPK1A'	mRNA	-0.837541231	7.316184456
54815	'GATAD2A'	mRNA	-0.823102571	49.62838549

9510	'ADAMTS1'	mRNA	-0.815574241	31.53299454
3975	'LHX1'	mRNA	-0.811525839	8.743104916
9674	'KIAA0040'	mRNA	-0.811118245	21.41988339
10379	'IRF9'	mRNA	-0.807660497	4.895317355
2533	'FYB1'	mRNA	-0.806878491	2.190710327
53834	'FGFRL1'	mRNA	-0.801873842	5.722456464
8864	'PER2'	mRNA	-0.800721003	5.00589134
6280	'S100A9'	mRNA	-0.794869242	2.040030542
5756	'TWF1'	mRNA	-0.78161878	75.00944131
8795	'TNFRSF10B'	mRNA	-0.779992944	56.89131641
4323	'MMP14'	mRNA	-0.777508607	53.00890118
55107	'ANO1'	mRNA	-0.774331451	15.21633626
26278	'SACS'	mRNA	-0.773554822	36.14036761
100133941	'CD24'	mRNA	-0.771449682	1.78990128
4939	'OAS2'	mRNA	-0.761964719	9.967735236
2039	'DMTN'	mRNA	-0.760119617	3.471771196
55802	'DCP1A'	mRNA	-0.76011234	17.27163105
26472	'PPP1R14B'	mRNA	-0.748315573	4.774323225
2810	'SFN'	mRNA	-0.747847998	67.90331822
145864	'HAPLN3'	mRNA	-0.743782599	1.814148386
8676	'STX11'	mRNA	-0.743295267	1.305592297
9644	'SH3PXD2A'	mRNA	-0.736759922	4.945485915
51042	'ZNF593'	mRNA	-0.736588728	7.561667468
79413	'ZBED2'	mRNA	-0.735641057	6.064344914
152519	'NIPAL1'	mRNA	-0.733839437	9.363440503
90161	'HS6ST2'	mRNA	-0.729914415	22.92684061
2119	'ETV5'	mRNA	-0.724211821	29.7082715
3164	'NR4A1'	mRNA	-0.723429795	2.214606352
7020	'TFAP2A'	mRNA	-0.722975013	13.09862867
8091	'HMGA2'	mRNA	-0.719582389	59.9901589
113146	'AHNAK2'	mRNA	-0.716465935	5.532877963
9962	'SLC23A2'	mRNA	-0.716431927	22.24277298
10468	'FST'	mRNA	-0.714679502	40.82460028
3872	'KRT17'	mRNA	-0.709706814	39.5515447
1969	'EPHA2'	mRNA	-0.708765622	63.8438575
54780	'NSMCE4A'	mRNA	-0.705893121	16.58291447
129607	'CMPK2'	mRNA	-0.705305755	1.987811837
80380	'PDCD1LG2'	mRNA	-0.703815093	6.561709017
154467	'CCDC167'	mRNA	-0.703689226	3.554587755
79817	'MOB3B'	mRNA	-0.702563674	6.88155717
5349	'FXYD3'	mRNA	-0.695809558	8.702317061
1902	'LPAR1'	mRNA	-0.695020883	16.00711335
8204	'NRIP1'	mRNA	-0.694435039	14.9741527
571	'BACH1'	mRNA	-0.694069683	25.66480035
60489	'APOBEC3G'	mRNA	-0.69384966	19.68000165
79094	'CHAC1'	mRNA	-0.689739216	7.059895918

80853	'KDM7A'	mRNA	-0.685481848	4.114880619
91107	'TRIM47'	mRNA	-0.68381201	10.91638048
65078	'RTN4R'	mRNA	-0.682489646	2.262772946
124491	'TMEM170A'	mRNA	-0.677270108	24.33194305
5530	'PPP3CA'	mRNA	-0.669064634	12.04303659
54438	'GFOD1'	mRNA	-0.667299626	7.823514802
4502	'MT2A'	mRNA	-0.6601266	53.54087551
118932	'ANKRD22'	mRNA	-0.644977713	4.923191726
2707	'GJB3'	mRNA	-0.644608714	22.25853133
257068	'PLCXD2'	mRNA	-0.643267231	7.264932295
94240	'EPSTI1'	mRNA	-0.638259352	4.924423875
150290	'DUSP18'	mRNA	-0.636912667	2.414458097
55357	'TBC1D2'	mRNA	-0.63158582	25.79663589
122553	'TRAPPC6B'	mRNA	-0.627034405	12.76361716
8519	'IFITM1'	mRNA	-0.625176268	1.703328729
118429	'ANTXR2'	mRNA	-0.624298248	14.03542124
23150	'FRMD4B'	mRNA	-0.623524629	2.077991044
3675	'ITGA3'	mRNA	-0.623288818	44.11712755
160897	'GPR180'	mRNA	-0.620907414	19.77584392
30001	'ERO1A'	mRNA	-0.619626123	41.67669345
182	'JAG1'	mRNA	-0.617967077	14.31169252
90120	'TMEM250'	mRNA	-0.617631825	14.36186477
9709	'HERPUD1'	mRNA	-0.617501383	13.79685955
3691	'ITGB4'	mRNA	-0.613747313	28.27203298
57458	'TMCC3'	mRNA	-0.613199165	7.674718293
54819	'ZCCHC10'	mRNA	-0.60928949	9.018384977
390928	'ACP7'	mRNA	-0.608868897	6.08453496
81848	'SPRY4'	mRNA	-0.60829939	6.824980721
5888	'RAD51'	mRNA	-0.607072052	10.05748429
259230	'SGMS1'	mRNA	-0.605275403	17.0561451
3213	'HOXB3'	mRNA	-0.604160386	1.751613336
100137047	'JMJD7'	mRNA	-0.603781648	1.425175156
55716	'LMBR1L'	mRNA	-0.602538274	3.492900447
3909	'LAMA3'	mRNA	-0.602236289	29.50040881
9107	'MTMR6'	mRNA	-0.602010794	21.97093841
331	'XIAP'	mRNA	-0.6012118	17.32657274
3976	'LIF'	mRNA	-0.599434228	29.88307582
154810	'AMOTL1'	mRNA	-0.595740912	28.11463259
595	'CCND1'	mRNA	-0.593557016	64.04712416
375449	'MAST4'	mRNA	-0.592757712	7.112961961
55638	'SYBU'	mRNA	-0.591289895	4.840956622
200316	'APOBEC3F'	mRNA	-0.584774308	4.924423875
57185	'NIPAL3'	mRNA	-0.581228134	20.5209231
994	'CDC25B'	mRNA	-0.581209812	15.42711598
1808	'DPYSL2'	mRNA	-0.58081318	1.398096792
1647	'GADD45A'	mRNA	-0.578889181	11.49236187

1475	'CSTA'	mRNA	-0.577338419	13.32866087
127544	'RNF19B'	mRNA	-0.577225681	9.583082334
1839	'HBEGF'	mRNA	-0.57187903	18.9372227
100128553	'CTAGE4'	mRNA	-0.571014189	2.427940013
101	'ADAM8'	mRNA	-0.569792871	36.41645867
8626	'TP63'	mRNA	-0.56833878	18.01726509
23052	'ENDOD1'	mRNA	-0.568322243	4.985192537
3726	'JUNB'	mRNA	-0.566042314	9.45459469
22862	'FNDC3A'	mRNA	-0.565695561	17.783489
7436	'VLDLR'	mRNA	-0.565311841	2.125225668
154141	'MBOAT1'	mRNA	-0.56406354	3.579171547
1019	'CDK4'	mRNA	-0.563110944	33.33594749
51804	'SIX4'	mRNA	-0.562573211	12.75171649
81706	'PPP1R14C'	mRNA	-0.562012173	2.433989322
25792	'CIZ1'	mRNA	-0.560557757	24.30540583
4856	'CCN3'	mRNA	-0.558989291	1.485688903
3866	'KRT15'	mRNA	-0.558709752	1.609541814
8462	'KLF11'	mRNA	-0.556872681	3.678670684
9980	'DOP1B'	mRNA	-0.556852291	2.904803052
822	'CAPG'	mRNA	-0.555728566	4.074606731
5328	'PLAU'	mRNA	-0.553561009	43.19768795
7378	'UPP1'	mRNA	-0.553265438	4.635241573
8850	'KAT2B'	mRNA	-0.55187297	1.582696675
54432	'YIPF1'	mRNA	-0.550364399	7.763519615
83595	'SOX7'	mRNA	-0.549941317	11.01826393
283	'ANG'	mRNA	-0.548520744	1.543531964
1601	'DAB2'	mRNA	-0.548292422	5.372906676
23432	'GPR161'	mRNA	-0.548089363	2.172030507
1825	'DSC3'	mRNA	-0.54700362	8.338984309
10184	'LHFPL2'	mRNA	-0.546043077	9.911525925
64393	'ZMAT3'	mRNA	-0.545453459	9.438446313
54585	'LZTFL1'	mRNA	-0.545412772	5.270236117
26160	'IFT172'	mRNA	-0.544887278	2.320431744
392	'ARHGAP1'	mRNA	-0.544075354	10.72089785
55970	'GNG12'	mRNA	-0.543055009	38.16312645
6509	'SLC1A4'	mRNA	-0.5421642	6.821943771
6773	'STAT2'	mRNA	-0.539605295	18.61543031
85441	'HELZ2'	mRNA	-0.539244507	7.551752743
4688	'NCF2'	mRNA	-0.53494883	2.89901814
90411	'MCFD2'	mRNA	-0.530350108	42.2031875
30837	'SOCS7'	mRNA	-0.528553777	11.22730171
160	'AP2A1'	mRNA	-0.528113504	13.82657991
966	'CD59'	mRNA	-0.527739812	56.09555039
440026	'TMEM41B'	mRNA	-0.527242104	8.370823586
7280	'TUBB2A'	mRNA	-0.524185345	5.372906676
55152	'DALRD3'	mRNA	-0.523518379	1.4266017

121274	'ZNF641'	mRNA	-0.52265134	2.012567999
200576	'PIKFYVE'	mRNA	-0.522003909	10.54544991
26511	'CHIC2'	mRNA	-0.521287793	2.804031047
23568	'ARL2BP'	mRNA	-0.518007465	15.25413744
83715	'ESPN'	mRNA	-0.513242948	1.453958795
3313	'HSPA9'	mRNA	-0.511939799	39.85141297
4600	'MX2'	mRNA	-0.511704431	1.922125232
100529063	'BCL2L2-PABPN1'	mRNA	-0.511269581	2.183631041
3936	'LCP1'	mRNA	-0.510881651	24.46687683
192670	'AGO4'	mRNA	-0.510814551	4.078307878
79712	'GTDC1'	mRNA	-0.510726814	4.827704262
11275	'KLHL2'	mRNA	-0.510110907	6.707670621
780	'DDR1'	mRNA	-0.508997779	21.08057157
57451	'TENM2'	mRNA	-0.508668291	4.575276022
81565	'NDEL1'	mRNA	-0.50826526	9.823998894
118472	'ZNF511'	mRNA	-0.508057008	6.472448923
5724	'PTAFR'	mRNA	-0.507926089	6.973318275
3297	'HSF1'	mRNA	-0.505628129	11.33897034
9168	'TMSB10'	mRNA	-0.504558636	25.57416153
928	'CD9'	mRNA	-0.503580996	16.00711335
8073	'PTP4A2'	mRNA	-0.502799465	33.27886468
10092	'ARPC5'	mRNA	-0.50120019	27.81662683
302	'ANXA2'	mRNA	-0.50102419	61.93924558
55601	'DDX60'	mRNA	-0.500793095	4.516973347
55741	'EDEM2'	mRNA	-0.500739678	5.797696534
10723	'SLC12A7'	mRNA	0.506015151	24.24890194
27018	'BEX3'	mRNA	0.50661787	7.513378164
3575	'IL7R'	mRNA	0.506699056	23.7426164
7127	'TNFAIP2'	mRNA	0.511341559	17.18485134
51200	'CPA4'	mRNA	0.512664588	2.372613835
3122	'HLA-DRA'	mRNA	0.514185364	13.12432118
84940	'CORO6'	mRNA	0.515929179	1.761332242
80008	'TMEM156'	mRNA	0.517853049	1.8565792
23048	'FNBP1'	mRNA	0.518993221	4.439145415
1906	'EDN1'	mRNA	0.52521863	12.39908331
7477	'WNT7B'	mRNA	0.526764716	13.79685955
7439	'BEST1'	mRNA	0.526913766	3.702712738
5563	'PRKAA2'	mRNA	0.532611662	2.060469109
2048	'EPHB2'	mRNA	0.536989607	10.65015473
400569	'MED11'	mRNA	0.5383463	2.028662226
22927	'HABP4'	mRNA	0.539285953	1.788920601
6988	'TCTA'	mRNA	0.540228844	2.849055745
414149	'ACBD7'	mRNA	0.545574539	2.063933813
144717	'PHETA1'	mRNA	0.546043991	1.615098134
112399	'EGLN3'	mRNA	0.55056982	5.699946206

2730	'GCLM'	mRNA	0.551980876	21.63585651
8844	'KSR1'	mRNA	0.553838338	1.509048999
245806	'VGLL2'	mRNA	0.554142485	6.886350503
93082	'NEURL3'	mRNA	0.556060401	1.443496383
123811	'CEP20'	mRNA	0.556911908	23.86970127
8470	'SORBS2'	mRNA	0.559659392	3.12649562
3127	'HLA-DRB5'	mRNA	0.560345287	1.665226173
80139	'ZNF703'	mRNA	0.563868356	1.68349657
64759	'TNS3'	mRNA	0.565327315	31.22675268
6648	'SOD2'	mRNA	0.565383142	40.69769582
113622	'ADPRHL1'	mRNA	0.567176377	1.518865496
6535	'SLC6A8'	mRNA	0.568977294	13.3998736
27076	'LYPD3'	mRNA	0.574557365	3.33715049
79669	'C3orf52'	mRNA	0.578386082	7.167733383
51523	'CXXC5'	mRNA	0.579051722	1.543037416
9183	'ZW10'	mRNA	0.579083862	6.023424275
9351	'NHERF2'	mRNA	0.588892033	9.579001893
9666	'DZIP3'	mRNA	0.589300997	3.159946694
1298	'COL9A2'	mRNA	0.589744753	1.443180569
348235	'SKA2'	mRNA	0.591000257	21.99011347
10397	'NDRG1'	mRNA	0.593560246	2.83112867
57561	'ARRDC3'	mRNA	0.593626501	8.708090016
2444	'FRK'	mRNA	0.593931081	7.841749854
727851	'RGPD8'	mRNA	0.59493753	1.880178233
84675	'TRIM55'	mRNA	0.605420111	14.21789349
254778	'VXN'	mRNA	0.607901024	2.051528037
25822	'DNAJB5'	mRNA	0.613874491	1.926584659
9315	'NREP'	mRNA	0.616658178	1.383003945
10333	'TLR6'	mRNA	0.621897495	2.199864021
3569	'IL6'	mRNA	0.623464903	12.1438694
10501	'SEMA6B'	mRNA	0.624719682	7.073820789
64081	'PBLD'	mRNA	0.624874573	1.337789093
2246	'FGF1'	mRNA	0.62590912	1.778009344
144165	'PRICKLE1'	mRNA	0.630815238	2.376261856
5468	'PPARG'	mRNA	0.634833467	2.948267916
22989	'MYH15'	mRNA	0.640431124	5.596176999
79092	'CARD14'	mRNA	0.641994571	1.604400307
374286	'FBXW10B'	mRNA	0.644591302	4.503994971
54537	'SHLD2'	mRNA	0.645377608	21.23724774
65999	'LRRC61'	mRNA	0.645906938	7.846739104
2634	'GBP2'	mRNA	0.64649119	4.402066009
3108	'HLA-DMA'	mRNA	0.646719514	2.91260497
10272	'FSTL3'	mRNA	0.652472781	4.784568286
768	'CA9'	mRNA	0.65544107	2.776519606
1021	'CDK6'	mRNA	0.657431289	44.07190743
4199	'ME1'	mRNA	0.657634364	29.01875257

64063	'PRSS22'	mRNA	0.668702148	7.692821857
4854	'NOTCH3'	mRNA	0.676228121	2.820183289
170960	'ZNF721'	mRNA	0.676561984	7.267125324
1031	'CDKN2C'	mRNA	0.67701988	5.541641933
7562	'ZNF708'	mRNA	0.678029064	1.381542339
624	'BDKRB2'	mRNA	0.681171246	2.199864021
80264	'ZNF430'	mRNA	0.682237882	8.527027054
5332	'PLCB4'	mRNA	0.682434846	2.843805087
343578	'ARHGAP40'	mRNA	0.683700267	1.47832178
7124	'TNF'	mRNA	0.685483105	7.432275651
2919	'CXCL1'	mRNA	0.687269055	43.1063173
2069	'EREG'	mRNA	0.690651348	13.59585834
283464	'GXYLT1'	mRNA	0.694193323	18.15347768
11240	'PADI2'	mRNA	0.698508653	3.767858328
3399	'ID3'	mRNA	0.705312565	5.893602748
23184	'MESD'	mRNA	0.705345893	29.70894857
85315	'PAQR8'	mRNA	0.717914571	2.015951681
22996	'TTC39A'	mRNA	0.719337808	2.190481155
90317	'ZNF616'	mRNA	0.723597653	3.201603908
8544	'PIR'	mRNA	0.725706507	10.10245073
2036	'EPB41L1'	mRNA	0.727818942	2.399823563
5218	'CDK14'	mRNA	0.728622229	7.488370978
80326	'WNT10A'	mRNA	0.7287552	4.781166889
4638	'MYLK'	mRNA	0.730367179	19.8974542
2678	'GGT1'	mRNA	0.731737798	5.210733279
629	'CFB'	mRNA	0.734434108	19.82837566
81544	'GDPD5'	mRNA	0.742065117	2.385395393
220963	'SLC16A9'	mRNA	0.742753188	4.149445495
80162	'PGGHG'	mRNA	0.743456166	23.99248233
117854	'TRIM6'	mRNA	0.745346666	3.516393384
55616	'ASAP3'	mRNA	0.745529988	1.988223745
8564	'KMO'	mRNA	0.757687155	4.711568155
8740	'TNFSF14'	mRNA	0.759109757	2.670197165
114907	'FBXO32'	mRNA	0.760434021	69.55257238
135	'ADORA2A'	mRNA	0.761988282	4.575391204
83729	'INHBE'	mRNA	0.762369118	1.608407082
283008	'NUTM2E'	mRNA	0.763503471	1.681131535
1942	'EFNA1'	mRNA	0.76717415	29.56057112
7047	'TGM4'	mRNA	0.770929895	2.102997577
117247	'SLC16A10'	mRNA	0.771645515	2.099758908
11259	'FILIP1L'	mRNA	0.774883135	8.861397394
23673	'STX12'	mRNA	0.77633325	27.39411863
80117	'ARL14'	mRNA	0.776597272	3.900321309
1545	'CYP1B1'	mRNA	0.777077867	3.779270623
54492	'NEURL1B'	mRNA	0.778825641	1.523310352
283234	'CCDC88B'	mRNA	0.785565981	8.55389996

9908	'G3BP2'	mRNA	0.786566763	56.37721741
199731	'CADM4'	mRNA	0.812999091	1.450328335
5806	'PTX3'	mRNA	0.81342663	20.66475064
54843	'SYTL2'	mRNA	0.819624358	1.42834424
1757	'SARDH'	mRNA	0.82113823	2.275517647
231	'AKR1B1'	mRNA	0.825787443	54.10472916
10396	'ATP8A1'	mRNA	0.828664122	3.351116663
51702	'PADI3'	mRNA	0.833715159	12.05642512
241	'ALOX5AP'	mRNA	0.837173131	7.799940086
1999	'ELF3'	mRNA	0.837702703	9.806574891
1294	'COL7A1'	mRNA	0.843521623	57.6937013
8862	'APLN'	mRNA	0.847670971	1.423273926
342132	'ZNF774'	mRNA	0.860745147	1.412164108
1759	'DNM1'	mRNA	0.865678115	7.484014125
7171	'TPM4'	mRNA	0.871616689	131.5353582
9723	'SEMA3E'	mRNA	0.879997717	1.947294215
10308	'ZNF267'	mRNA	0.88607231	15.85215259
5915	'RARB'	mRNA	0.892923946	1.493335853
4739	'NEDD9'	mRNA	0.897855542	6.500605298
114794	'ELFN2'	mRNA	0.917890249	2.496373
57216	'VANGL2'	mRNA	0.924348038	1.954616261
8997	'KALRN'	mRNA	0.934755247	3.167620292
5210	'PFKFB4'	mRNA	0.936000051	19.28182101
6347	'CCL2'	mRNA	0.940307374	92.54617893
29943	'PADI1'	mRNA	0.94156806	2.747768229
4261	'CIITA'	mRNA	0.947069825	19.28182101
200958	'MUC20'	mRNA	0.948867078	6.794104107
90427	'BMF'	mRNA	0.955670932	2.807651587
51704	'GPRC5B'	mRNA	0.958420158	27.11992203
840	'CASP7'	mRNA	0.980052163	45.71735731
23308	'ICOSLG'	mRNA	0.989806156	3.480037865
283209	'PGM2L1'	mRNA	0.990048775	25.90279645
9536	'PTGES'	mRNA	0.994124075	3.602311095
64718	'UNKL'	mRNA	0.994609256	34.51986295
5653	'KLK6'	mRNA	0.996386183	2.35895625
148206	'ZNF714'	mRNA	1.005049866	26.09645285
494470	'ARK2C'	mRNA	1.017699749	1.371254332
91683	'SYT12'	mRNA	1.018227546	7.032478728
55124	'PIWIL2'	mRNA	1.023366509	2.064226361
6236	'RRAD'	mRNA	1.026642938	4.279725319
116372	'LYPD1'	mRNA	1.028923724	1.826779564
57722	'IGDCC4'	mRNA	1.049894076	2.212249954
102723996	'LOC102723996'	mRNA	1.055950071	8.908890897
3955	'LFNG'	mRNA	1.061277307	11.68208608
64288	'ZSCAN31'	mRNA	1.069402279	2.851116024
83716	'CRISPLD2'	mRNA	1.094224555	11.35055656

1543	'CYP1A1'	mRNA	1.095760253	4.848931901
27134	'TJP3'	mRNA	1.096145	1.62128795
7113	'TMPRSS2'	mRNA	1.099073494	4.404082263
84171	'LOXL4'	mRNA	1.109319134	1.973749024
7697	'ZNF138'	mRNA	1.127747867	12.33593801
29113	'C6orf15'	mRNA	1.13465523	2.743636363
56062	'KLHL4'	mRNA	1.192239417	1.807801888
3576	'CXCL8'	mRNA	1.208936519	8.326644101
4973	'OLR1'	mRNA	1.23258689	20.88494685
6615	'SNAI1'	mRNA	1.242432057	2.010449104
23109	'DDN'	mRNA	1.248447304	1.450573198
1277	'COL1A1'	mRNA	1.267724695	10.02049948
345651	'ACTBL2'	mRNA	1.269391489	1.616436163
92737	'DNER'	mRNA	1.280940162	17.15510191
2775	'GNAO1'	mRNA	1.311391473	1.926584659
2925	'GRPR'	mRNA	1.345856495	3.874099781
2318	'FLNC'	mRNA	1.364652101	3.594923253
1295	'COL8A1'	mRNA	1.371621392	1.649296214
3589	'IL11'	mRNA	1.383693328	26.91277182
563	'AZGP1'	mRNA	1.397218166	1.436717942
1755	'DMBT1'	mRNA	1.443632871	11.01942822
116154	'PHACTR3'	mRNA	1.479775441	1.717747943
10156	'RASA4'	mRNA	1.503027312	2.543186058
1917	'EEF1A2'	mRNA	1.505983663	7.87345093
23604	'DAPK2'	mRNA	1.52096285	1.96141074
282973	'JAKMIP3'	mRNA	1.683331092	3.738687372
4050	'LTB'	mRNA	1.881753038	7.033750086
257106	'ARHGAP30'	mRNA	1.973873855	1.872122463
101060351	'TBC1D3K'	mRNA	2.046064069	1.4004548
1734	'DIO2'	mRNA	2.465108856	7.698441724
79919	'MAB21L4'	mRNA	3.292395921	19.94507497
100527943	'TGIF2-RAB5IF'	mRNA	4.039066367	1.417766656
